# Effect of Wearing a Face Mask on Hand-to-Face Contact by Children in a Simulated School Environment: The Back-to-School COVID-19 Simulation Randomized Clinical Trial

**DOI:** 10.1001/jamapediatrics.2022.3833

**Published:** 2022-10-24

**Authors:** Michelle Science, Monica Caldeira-Kulbakas, Rulan S. Parekh, Bryan R. Maguire, Stacie Carroll, Samantha J. Anthony, Ari Bitnun, Laura E. Bourns, Douglas M. Campbell, Eyal Cohen, Alison Dodds, Vinita Dubey, Jeremy N. Friedman, Jodi L. Greenwood, Jessica P. Hopkins, Ryan Imgrund, Daphne J. Korczak, Thomas Looi, Emily Louca, Dominik Mertz, John Nashid, Giovanna Panzera, Jane E. Schneiderman, Kevin L. Schwartz, Laurie Streitenberger, Sunayna Vuppal, Catharine M. Walsh, Peter Jüni, Clyde T. Matava

**Affiliations:** 1 Division of Infectious Diseases, Department of Paediatrics, The Hospital for Sick Children, Toronto, Ontario, Canada; 2 Public Health Ontario, Toronto, Ontario, Canada; 3 Department of Paediatrics, Faculty of Medicine, University of Toronto, Toronto, Ontario, Canada; 4 Department of Anesthesia and Pain Medicine, The Hospital for Sick Children, Toronto, Ontario, Canada; 5 Child Health Evaluative Sciences, The Hospital for Sick Children, Toronto, Ontario, Canada; 6 Division of Nephrology, Department of Pediatrics and Medicine, The Hospital for Sick Children, Toronto, Ontario, Canada; 7 Biostatistics Design and Analysis Unit, Research Institute, The Hospital for Sick Children, Toronto, Ontario, Canada; 8 Child and Family Centred Care, The Hospital for Sick Children, Toronto, Ontario, Canada; 9 Education and Community Partnership Program, Toronto District School Board, Toronto, Ontario, Canada; 10 Factor-Inwentash Faculty of Social Work, University of Toronto, Toronto, Ontario, Canada; 11 Neonatal Intensive Care Unit, St Michael's Hospital, Unity Health Toronto, Toronto, Ontario, Canada; 12 Allan Waters Family Simulation Program, St Michael's Hospital, Unity Health Toronto, Toronto, Ontario, Canada; 13 Li Ka Shing Knowledge Institute, Unity Health Toronto, Toronto, Ontario, Canada; 14 Complex Care Program, Department of Paediatrics, The Hospital for Sick Children, Toronto, Ontario, Canada; 15 Institute of Health Policy, Management & Evaluation, University of Toronto, Toronto, Ontario, Canada; 16 Edwin S.H. Leong Centre for Healthy Children, University of Toronto, Toronto, Ontario, Canada; 17 SimKids Simulation Program, The Learning Institute, The Hospital for Sick Children, Toronto, Ontario, Canada; 18 Communicable Disease Control, Toronto Public Health, Toronto, Ontario, Canada; 19 Dalla Lana School of Public Health, University of Toronto, Toronto, Ontario, Canada; 20 Department of Paediatrics, The Hospital for Sick Children, Toronto, Ontario, Canada; 21 Department of Health Research Methods, Evidence, and Impact, McMaster University, Hamilton, Ontario, Canada; 22 Biostatistics, Southlake Regional Health Centre, Newmarket, Ontario, Canada; 23 Department of Psychiatry, The Hospital for Sick Children, Toronto, Ontario, Canada; 24 Department of Psychiatry, University of Toronto, Toronto, Ontario, Canada; 25 Department of Neuroscience and Mental Health, The Hospital for Sick Children, Toronto, Ontario, Canada; 26 The Wilfred and Joyce Posluns Centre for Image-Guided Innovation and Therapeutic Intervention, The Hospital for Sick Children, Toronto, Ontario, Canada; 27 Department of Medicine, McMaster University, Hamilton, Ontario, Canada; 28 Department of Infection Prevention and Control, Hamilton Health Sciences, Hamilton, Ontario, Canada; 29 Corporate Strategy, The Hospital for Sick Children, Toronto, Ontario, Canada; 30 Division of Respiratory Medicine, Clinical Research Services, The Hospital for Sick Children, Toronto, Ontario, Canada; 31 Faculty of Kinesiology and Physical Education, University of Toronto, Toronto, Ontario, Canada; 32 Division of Infectious Diseases, Department of Medicine, Unity Health Toronto, Toronto, Ontario, Canada; 33 Infection Prevention & Control (IPAC) Program, The Hospital for Sick Children, Toronto, Ontario, Canada; 34 Division of Gastroenterology, Hepatology and Nutrition, Department of Paediatrics, The Hospital for Sick Children, Toronto, Ontario, Canada; 35 Applied Health Research Centre, Li Ka Shing Knowledge Institute of St Michael's Hospital, Toronto, Ontario, Canada; 36 Department of Medicine, University of Toronto, Toronto, Ontario, Canada; 37 Department of Anesthesiology and Pain Medicine, Faculty of Medicine, University of Toronto, Toronto, Ontario, Canada

## Abstract

**Importance:**

Wearing a face mask in school can reduce SARS-CoV-2 transmission but it may also lead to increased hand-to-face contact, which in turn could increase infection risk through self-inoculation.

**Objective:**

To evaluate the effect of wearing a face mask on hand-to-face contact by children while at school.

**Design, Setting, and Participants:**

This prospective randomized clinical trial randomized students from junior kindergarten to grade 12 at 2 schools in Toronto, Ontario, Canada, during August 2020 in a 1:1 ratio to either a mask or control class during a 2-day school simulation. Classes were video recorded from 4 angles to accurately capture outcomes.

**Interventions:**

Participants in the mask arm were instructed to bring their own mask and wear it at all times. Students assigned to control classes were not required to mask at any time (grade 4 and lower) or in the classroom where physical distancing could be maintained (grade 5 and up).

**Main Outcomes and Measures:**

The primary outcome was the number of hand-to-face contacts per student per hour on day 2 of the simulation. Secondary outcomes included hand-to-mucosa contacts and hand-to-nonmucosa contacts. A mixed Poisson regression model was used to derive rate ratios (RRs), adjusted for age and sex with a random intercept for class with bootstrapped 95% CIs.

**Results:**

A total of 174 students underwent randomization and 171 students (mask group, 50.6% male; control group, 52.4% male) attended school on day 2. The rate of hand-to-face contacts did not differ significantly between the mask and the control groups (88.2 vs 88.7 events per student per hour; RR, 1.00; 95% CI, 0.78-1.28; *P* = >.99). When compared with the control group, the rate of hand-to-mucosa contacts was significantly lower in the mask group (RR, 0.12; 95% CI, 0.07-0.21), while the rate of hand-to-nonmucosa contacts was higher (RR, 1.40; 95% CI, 1.08-1.82).

**Conclusions and Relevance:**

In this clinical trial of simulated school attendance, hand-to-face contacts did not differ among students required to wear face masks vs students not required to wear face masks; however, hand-to-mucosa contracts were lower in the face mask group. This suggests that mask wearing is unlikely to increase infection risk through self-inoculation.

**Trial Registration:**

ClinicalTrials.gov Identifier: NCT04531254

## Key Points

**Question:**

Does wearing a face mask lead to increased hand-to-face contact in children?

**Findings:**

In this randomized clinical trial of 174 children aged 5 to 18 years, the rate of hand-to-face contact was not significantly different between children wearing a face mask and the control group in a simulated school environment.

**Meaning:**

Face mask wearing did not increase hand-to-face or hand-to-mucosa contact in children, suggesting that mask wearing is unlikely to increase infection risk through self-inoculation.

## Teaser

This randomized clinical trial evaluates the effect of wearing a face mask on hand-to-face contact by children while at school.

## Introduction

The use of face masks in community settings is an integral part of a layered approach of public health measures used to reduce severe acute respiratory syndrome coronavirus 2 (SARS-CoV-2) transmission and the incidence of COVID-19.^1,2,3,4,5^ However, face mask use in children and schools remains controversial, with varying jurisdictional approaches reflecting how stakeholders weigh the potential benefits and negative consequences.^6^ The benefits include source control and protection of the wearer, depending on the material used and the fit of the mask.^5,6,7^

Several studies have demonstrated low secondary attack rates in schools where masking was one of the health and safety measures in place.^8,9,10,11,12,13^ In addition, epidemiologic studies have shown fewer outbreaks^14^ and lower incidence of SARS-CoV-2 infection^15^ in areas with school masking policies. However, these benefits need to be evaluated along with the potential negative consequences. Potential negative consequences include physical adverse effects (respiratory and dermatologic) and psychological, cognitive, and communicative effects.^16,17^

It has been suggested that wearing a face mask could lead to increased hand-to-face contact in children, which could result in self-inoculation and transmission of viruses.^18,19,20,21^ Therefore, we conducted the Back-to-School COVID-19 Simulation Trial to evaluate the effects of wearing a face mask on hand-to-face contact by children in a simulated school environment.

## Methods

### Trial Oversight

The trial was an investigator-initiated, open-label, randomized clinical trial of face masks involving students from junior kindergarten (JK) to grade 12 (4 to 17 years old) who participated in a simulated school environment for 2 consecutive days. The trial was conducted at 2 schools in Toronto, Ontario, Canada, in August 2020, at a time when schools were closed for the summer break after a prolonged closure to in-person learning related to the COVID-19 pandemic. Ethical approval was obtained from the Hospital for Sick Children’s research ethics board (REB # 1000071861). Even though the trial was based on a school simulation and did not assess health outcomes, it was registered with ClinicalTrials.org (NCT04531254). The protocol is available with the full text of this article online. There were no significant changes to the Methods after trial commencement (eMethods in Supplement 1). Written informed consent of parents/caregivers or children and verbal assent of all children when parents/caregivers provided consent were obtained for all participants.

The trial protocol design team consisted of pediatricians, infectious disease specialists, infection prevention and control professionals, epidemiologists, medical simulation experts, and schoolteachers. The investigators vouch for the completeness and accuracy of the data and analyses, and for the fidelity of the trial to the protocol and statistical analysis plan. The first draft of the article was written by the first and last authors according to the Consolidated Standards of Reporting Trials (CONSORT) reporting guidelines^22^ and revised by all authors, who agreed to submit the article for publication.

### Participants

Participants were recruited from the greater Toronto area using social media, news releases, and direct communication from school principals (eMethods in Supplement 1). Students were eligible if they attended school or a structured learning environment during the 2019 to 2020 school year and self-reported that they did not require additional support beyond those provided by a single class teacher. Teachers were eligible if they were certified by the Ontario College of Teachers. Students and teachers were excluded if they had risk factors for SARS-CoV-2 infection (eMethods in Supplement 1) or a known hypersensitivity or allergy to a biological indicator used in the trial (Glo Germ; Glo Germ Company). All participants had a negative SARS-CoV-2 polymerase chain reaction test result by nasopharyngeal swab in the 48 hours before the start of the study.

### Intervention

Students and teachers were assigned in a 1:1 ratio to either mask or control classes in 1 of 7 grade ranges (kindergarten, grades 1 and 2, grades 3 and 4, grades 5 and 6, grades 7 and 8, grades 9 and 10, and grades 11 and 12) based on the grade they had just completed (students) or had teaching experience (teachers). Students assigned to mask classes were instructed to bring their own mask and always wear it, even when distancing could be maintained, except when eating and drinking. Students assigned to control classes were told that they did not need to wear a mask at all (grades 4 and lower) or did not need to wear a mask when physical distancing (2 m or more) could be maintained (grades 5 and up), but masking was allowed. Pediatric masks were available for students in the masking class who forgot to bring a mask or needed a replacement. Teachers were provided with medical masks and wore the mask irrespective of whether they were teaching a mask or control class. Classrooms were set up to allow physical distancing of 2 m or more between desks, such that grades 5 and up did not need to wear masks when at their desk in the classroom, consistent with provincial public health measures at the time.

### Trial Procedures

Two full school days (8:35 am to 3 pm) were planned after randomization. On the first day, students adjusted to return to school procedures, including mask use if randomized to the mask class. On the second day, the same procedures were followed, and outcomes were assessed. Teachers developed the curriculum to simulate a regular school day. Policies and procedures were put in place to replicate the provincial plans for return to school in September 2020.^23^ The curriculum and procedure are outlined in the eMethods in Supplement 1.

### Outcomes

The primary outcome was the number of hand-to-face contacts per student per hour on the second day of the simulation. We defined a hand-to-face contact as starting from the moment a hand touched the face, until the hand lifted off the face. Classrooms were recorded using a secure closed-circuit television setup on a closed-wired area network to capture information on primary and secondary outcomes. Four cameras were set up in each classroom to capture all angles (eFigure 1 in Supplement 1). Two 60-minute time periods (1 in the morning, 1 after lunch) were used to assess outcomes when students were present in the classroom participating in classroom activities (excluding lunch). The details on video data acquisition and storage are provided in the eMethods in Supplement 1. For each grade range, the occurrence and type of hand-to-face contacts were recorded by a single trained coder who reviewed all the video footage in both mask and control classes during class time (excluding periods of eating and drinking). Hand-to-face-contacts were classified for each hand as contact with the (1) mouth and/or nose, (2) eyes, (3) glasses, (4) other nonmucosa part of the face (eg, chin, ears, cheek, forehead), (5) central mask (over mouth/nose), (6) peripheral mask (side of mask/ear loops), (7) removing mask, and (8) putting on mask. When there were multiple areas touched during 1 contact, coders were instructed to prioritize mucosa (codes 1 or 2) over nonmucosa codes (codes 3 to 8). Within nonmucosa touches, mask touches (codes 5 to 8) took precedence over other face contacts (code 4). The primary outcome included codes 1 to 8. Touches were time-stamped by hour, minute, and second (when multiple touches occurred within a minute). The prespecified key secondary outcomes were hand-to-mucosa contacts (codes 1 and 2), and hand-to-nonmucosa face contacts, including mask (codes 3 to 8), per student per hour. The additional prespecified secondary outcomes included the numbers of interpersonal hand holding per student per hour, the number of mask removals per student per hour, and the numbers of students and teachers with biotracer contamination. Five of 12 secondary outcomes could not be assessed for logistical or technical reasons (eMethods in Supplement 1), whereas experiences of students and teachers were examined in 2 substudies, and have been reported separately.^24,25^ Two outcomes were not prespecified in the protocol, but analyzed posthoc: the number of hand-to-mask contacts (codes 5 to 8) and the number of other nonmucosa face contacts (code 4) per student per hour.

Coders received training by a single trainer (M.C.K.) on how to code hand-to-face-contacts using archive videos and underwent an assessment to ensure consistency of coding of at least 10 minutes of video footage in comparison with the trainer (eMethods in Supplement 1). To enable an interrater reliability study of the primary and the 2 key secondary outcomes, we randomly selected a period of 10 minutes during class time for each class. During this period, these outcomes were independently assessed by a second coder for each student.

### Randomization

After inclusion of all participants, central randomization was done on the day before the start of the simulation by an independent statistician based on computer-generated randomization schedules stratified by age and sex for students and by range of grades for teachers. Randomization was blocked, with randomly varied block sizes for students and blocks of 2 for teachers. Allocation was communicated to parents and students immediately after randomization on the day before the start of the simulation. In addition, students and teachers were kept unaware of the specific objectives of the trial, including the prespecified primary outcome (hand-to-face contact) and all key secondary outcomes.

### Statistical Analysis

We hypothesized that mask use would increase the rate of hand-to-face contacts. Assuming an intracluster correlation coefficient of 0.01 for the correlation of hand-to-face contacts of students within classes, a standard deviation of the number of hand-to-face contacts per hour per student of 6, and a class size of 15 students, we estimated that 12 classes with 180 students would result in more than 80% power to detect an increase in the number of hand-to-face contacts per hour per student from an average of 30 in control classes to an average of 33 in mask classes, corresponding to a rate ratio of 1.1, at a 2-sided α of .05. This difference of 3 contacts per hour was determined to be a clinically relevant treatment effect based on expert opinion and modeling studies on the impact of hand-to-face contacts on infection risk.^26,27^ The trial was adaptive using a sequential design with an interim analysis planned after the first 2 days of school simulation, and was stopped because the 2-sided *z* of 0 crossed the boundary of equivalence (eFigure 2 in Supplement 1). Analyses were performed in the modified intention-to-treat population, which included all randomly assigned students and teachers who participated on the second day of the simulation.

Primary and secondary analyses used a mixed Poisson regression model adjusted for age and sex with a random intercept for class to derive rate ratios, with bootstrapped 95% CIs and 2-sided *P* values. Subgroup analyses planned a priori included sex, self-reported ethnicity, school type (private vs public), grade (JK through grades 4 vs grades 5 through 12). Since the widths of 95% CIs for secondary outcomes were not adjusted for multiple comparisons, these intervals should not be used for inferences about treatment effects. Intraclass correlation coefficients^28^ and Bland Altman plots^29^ were used to determine interrater reliability. Sensitivity analyses were done after exclusion of students who adhered to the allocated intervention in less than 70% of class time. All analyses were performed with R, release 4.0.5 (R Foundation).

## Results

### Participants

**Figure 1.**
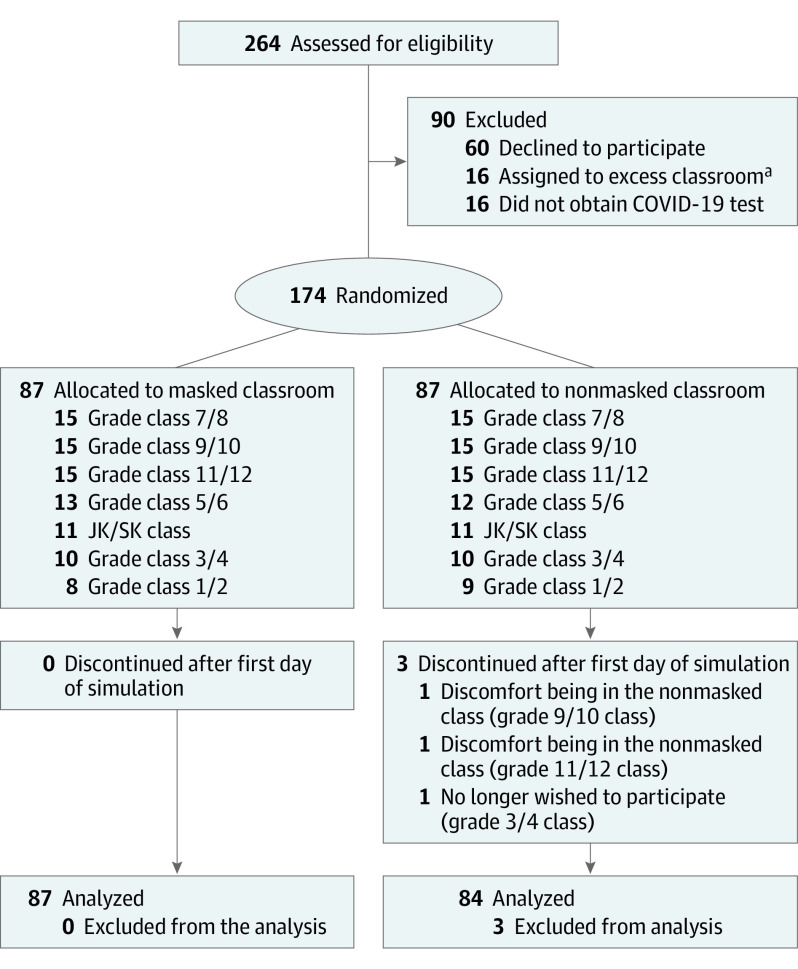
CONSORT Diagram ^a^Class sizes were capped at 15 students based on anticipated Ontario Ministry of Education guidelines for classroom distancing.

In August 2020, 264 students were assessed for eligibility, 174 students were deemed eligible, provided consent and assent, and 87 students were randomized to each group (Figure 1). In the mask group, all students completed the trial, whereas in the control group, 3 students withdrew after the first day and were lost to follow-up. The characteristics of the participants in the 2 groups were similar at baseline (Table 1). The median age was 12.4 (interquartile range, 9.1-15.2) years, 48.5% were female, 50.3% were White, and 61% attended a public school in the previous year. Overall, 87 students (100%) in the mask group and 82 students (94.3%) in the control group received the intervention as allocated; 2 students in the control group wore their mask 100% of time. Seventy-one students (81.6%) and 80 students (92.0%), respectively, adhered to the allocated intervention in more than 70% of class time on the second day of school simulation. A total of 171 students were included in the intention-to-treat analysis, 87 students in the mask group, and 84 students in the control group.

**Table 1.** Characteristics of the Students Participating in the Randomized School Simulation Trial by Group

Characteristic	No. (%)	
	Mask class (n = 87)	Control class (n = 84)
Age, median (IQR), y	12.3 (9.1-15.2)	12.5 (8.7-15.1)
Sex		
Female	43 (49.4)	40 (47.6)
Male	44 (50.6)	44 (52.4)
Public school	49 (58.3)	51 (63.8)
Self-reported ethnicity		
Asian	16 (18.4)	12 (14.3)
Black	7 (8.0)	5 (6.0)
White	46 (52.9)	40 (47.6)
Other/unknown^a^	18 (20.7)	27 (32.1)
Income, $		
≤99 999	14/82 (17.1)	11/80 (13.8)
100 000-199 999	11/82 (13.4)	23/80 (28.8)
≥200 000	44/82 (53.7)	39/80 (48.8)
Chose not to answer	13/82 (15.9)	7/80 (8.8)
Grade		
JK/SK	11 (12.6)	11 (13.1)
1-2	8 (9.2)	9 (10.7)
3-4	10 (11.5)	9 (10.7)
5-6	13 (14.9)	12 (14.3)
7-8	15 (17.2)	15 (7.9)
9-10	15 (17.2)	14 (16.7)
11-12	15 (17.2)	14 (16.7)

Abbreviations: IQR, interquartile range; JK, junior kindergarten; SK, senior kindergarten.

^a^ The individuals listed other without providing a written comment.

### Outcomes

**Figure 2.**
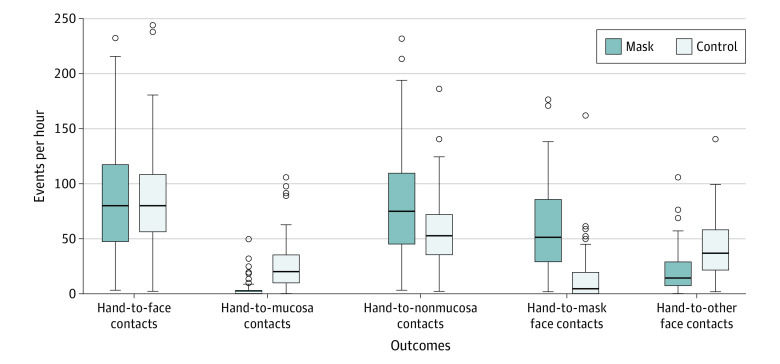
Distribution of Number of Hand-to-Face Contacts by Mask Group and Control Group

The rate of hand-to-face contacts did not differ significantly between groups (88.2 vs 88.7 events per hour in mask and control groups, respectively; rate ratio [RR], 1.00; 95% CI, 0.78-1.28; *P* = >.99) (Table 2). The rate of hand-to-mucosa contacts was significantly lower in the mask group (4.2 vs 26.8 events per hour; RR, 0.12; 95% CI, 0.07-0.21), while the rate of hand-to-nonmucosa contacts was higher in the mask group (84.4 vs 58.1 events per hour; RR, 1.40; 95% CI, 1.08-1.82) (Figure 2). The rate of hand-to-mask contacts was higher in the mask group (60.3 vs 14.2 events per hour; RR, 16.43; 95% CI, 2.49-103.73), whereas other face touches were less frequent (21.1 vs 42.7 events per hour; RR, 0.46; 95% CI, 0.33-0.65). There were no incidences of hand holding in either group. Masks were removed 102 times in the mask group (rate 0.6 events per student per hour) (Table 2).

**Table 2.** Adjusted Analyses of the Impact of Face Mask Wearing on the Primary and Secondary Outcomes in the Simulated School Environment

Outcome	No. of events (rate or risk)^a^		Rate ratio or risk ratio (95% CI)	*P* value
	Mask group	Control group		
Students, No.	87	84	NA	NA
Hours, No.	172.6	168.0	NA	NA
Hand-to-face contacts	15 224 (88.2)	14 560 (88.7)	1.00 (0.78-1.28)	>.99
Hand-to-mucosa contacts^b^	719 (4.2)	4503 (26.8)	0.12 (0.07-0.21)	NA
Hand-to-nonmucosa contacts	14 368 (84.4)	9757 (58.1)	1.40 (1.08-1.82)	NA
Hand-to-mask contacts^c^	10 401 (60.3)	2383 (14.2)	16.43 (2.49-103.73)	NA
Hand-to-other face contacts^c^	3635 (21.1)	7176 (42.7)	0.46 (0.33-0.65)	NA
Hand holding	0	0	NA	NA
Mask removals	102 (0.6)	NA	NA	NA

Abbreviation: NA, not applicable.

^a^ Rates are mean numbers of events per hour of observation time; risks are percentages.

^b^ Most of hand-to-mucosa contacts were mouth/nose contacts with no difference in eye contact between groups (eTable 3 in Supplement 1).

^c^ Posthoc analyses to demonstrate the breakdown of hand-to-nonmucosa contacts between groups.

The effect of the intervention on the primary outcome was consistent across prespecified subgroups, with no differences in effect estimates by age, sex, ethnicity, or school attendance (private vs public; eFigure 3 in Supplement 1). The intraclass correlation coefficients were 0.72 for the primary outcome (95% CI, 0.67-0.77), 0.83 for hand-to-mucosa touches (95% CI, 0.79-0.86), and 0.72 for hand-to-nonmucosa touches (95% CI, 0.66-0.77) (eTable 1 in Supplement 1). Bland-Altman plots are presented in eFigure 4 in Supplement 1. Sensitivity analyses after exclusion of students who adhered to the allocated intervention in 70% or less of class time showed similar results as primary analyses (eTable 2 in Supplement 1).

## Discussion

In this randomized school simulation clinical trial with video recordings to capture primary and secondary outcomes, wearing a face mask did not increase hand-to-face contacts and resulted in a decrease in hand-to-mucosa contacts. Findings were consistent for the primary outcome across subgroups defined by age, sex, ethnicity, and private or public school attendance.

To our knowledge, this is the first randomized trial evaluating the impact of masks on hand-to-face contacts and the first study in children. Using public video recordings, 2 retrospective studies in the general population either found no association between mask use and hand-to-face contacts^30^ or a decrease in hand-to-face contacts, especially hand-to-mucosa contacts.^31^ In a prospective cohort study in health care professionals, mask use was associated with reduced hand-to-face contacts.^32^ All 3 studies included only short observation times per individual (less than 1 minute for video observations^30,31^ and 5 to 15 minutes for in-person observation^32^). Our trial accumulated nearly 2 hours of observation time per student using video from 4 angles to accurately capture behaviors in a school setting, where behaviors may be different given the nature of interactions and environment.^33^

As face touching can lead to self-inoculation, the frequency of hand-to-face contacts is considered important and has been used in exposure modeling to quantify infectious dose.^34^ Rates previously described in adults ranged from 3.3 touches per hour (community)^35^ to 19 to 23 touches per hour (health care professionals^36^ and medical students^37^). In a prospective study without mask use, children were approximately 1.3 times more likely to touch their faces than adults.^38^ We therefore assumed 30 hand-to-face contacts per hour when designing our trial and were surprised to find nearly 3 times higher rates. This suggests an underestimation in previous studies using less sensitive methods for outcome ascertainment.

These findings have important public health implications. Wearing a face mask did not lead to increased hand-to-face contact, but were associated with reduced hand-to-mucosa contact, which may add protection. In addition, SARS-CoV-2 transmission through hand-to-mucosa contact is less likely than through inhalation.^39,40,41^ Taken together, this suggests that the benefits of masks for infection prevention in children clearly outweigh any potential infectious risks. Our finding of frequent face touching in children is important for the understanding of infection risk with other viruses. Hand-to-face contact is implicated in transmission by the fecal-oral route (eg, norovirus and other gastrointestinal pathogens) and has been suggested to contribute to transmission of respiratory rhinovirus,^19^ influenza,^20^ and beta coronaviruses.^42^ This emphasizes the importance of hand hygiene in schools.^43^

### Limitations

Our trial has several limitations. It was impossible to blind coders to treatment allocation. We provided the coders with standardized training, a verification process to ensure coding reliability prior to initiating the study coding, and demonstrated good interrater reliability of coding. Some students in the unmasked group wore masks for variable periods of time during the study period, such as periods of movement within the school. However, sensitivity analyses removing those participants that did not adhere to their allocation for more than 70% of the time did not demonstrate a difference in results. Additionally, our results may not be generalizable to children who require additional resources, for example, children with medical needs.

## Conclusions

In conclusion, in this clinical trial, wearing face masks in a simulated school environment did not result in more hand-to-face contacts. These findings suggest that face mask wearing by children in schools, it is unlikely to lead to increased infection risk through self-inoculation.

## Notes

Supplement 1. eMethods. eFigure 1. Closed-circuit Television camera setup for each classroom eFigure 2. Sequential analysis eFigure 3. Subgroup Analyses evaluating the impact of face mask wearing on the hand-to-face contact eFigure 4. Bland-Altman plots depicting agreement between pairs of coders on the study primary outcome eTable 1. Intraclass Correlation Coefficients for pairs of coders eTable 2. Sensitivity Analysis eTable 3. Breakdown of number of face or mask contact by code classification

Supplement 2. Original Protocol.

Supplement 3. Summary of Protocol Changes

Supplement 4. Final Protocol

Supplement 5. Nonauthor Collaborator

Supplement 6. Data Sharing Statement
