# Supplementary material for: Effect of Wearing a Face Mask on Hand-to-Face Contact by Children in a Simulated School Environment: The Back-to-School COVID-19 Simulation Randomized Clinical Trial
Source: JAMA Pediatr. 2022 Oct 24;176(12):1169–75. doi: 10.1001/jamapediatrics.2022.3833 (PMC9593317; doi:10.1001/jamapediatrics.2022.3833)
Supplement: Supplement 1. — eMethods. eFigure 1. Closed-circuit Television camera setup for each classroom eFigure 2. Sequential analysis eFigure 3. Subgroup Analyses evaluating the impact of face mask wearing on the hand-to-face contact eFigure 4. Bland-Altman plots depicting agreement between pairs of coders on the study primary outcome eTable 1. Intraclass Correlation Coefficients for pairs of coders eTable 2. Sensitivity Analysis eTable 3. Breakdown of number of face or mask contact by code classification [file jamapediatr-e223833-s001.pdf]

Supplemental Online Content

Science M, Caldeira-Kulbakas M, Parekh RS, et al; the Back to School COVID-19 School Study Group. Effect of wearing a face mask on hand-to-face contact by children in a simulated school environment: the Back-to-School COVID-19 simulation randomized clinical trial. *JAMA Pediatr*. Published online October 24, 2022. doi:10.1001/jamapediatrics.2022.3833

eMethods.
eFigure 1. Closed-circuit Television camera setup for each classroom
eFigure 2. Sequential analysis
eFigure 3. CONSORT Diagram for the trial
eFigure 4. Subgroup Analyses evaluating the impact of face mask wearing on the hand-to-face contact
eFigure 5. Bland-Altman plots depicting agreement between pairs of coders on the study primary outcome
eTable 1. Intraclass Correlation Coefficients for pairs of coders
eTable 2. Sensitivity Analysis
eTable 3. Breakdown of number of face or mask contact by code classification

This supplemental material has been provided by the authors to give readers additional information about their work.

eMethods

R Packages

The following packages were used in R: lme4[1], pbkrtest[2], and emmeans[3]. Citations are provided below.

1. Douglas Bates, Martin Maechler, Ben Bolker, Steve Walker (2015). Fitting Linear Mixed-Effects Models Using lme4. Journal of Statistical Software, 67(1), 1-48. doi:10.18637/jss.v067.i01.

2. Ulrich Halekoh, Søren Højsgaard (2014). A Kenward-Roger Approximation and Parametric Bootstrap Met hods for Tests in Linear Mixed Models - The R Package pbkrtest. Journal of Statistical Software, 59(9), 1-3 0. URL https://www.jstatsoft.org/v59/i09/.

3. Russell V. Lenth (2022). emmeans: Estimated Marginal Means, aka Least-Squares Means. R package versi on 1.7.2. https://CRAN.R-project.org/package=emmeans

Recruitment Details

Participants were recruited using email communication from school principals, and media and, news releases, and three public webinars. Interested participants were directed to the study website (https://safeschoolcovid19.ca/) where they could express willingness to participate via online form submission or by directly contacting the research team by phone or email.

Exclusion Criteria - Risk Factors for SARS-CoV-2 Infection

1) Exposed to an individual that tested positive for SARS-CoV-2 in the 14 days before the trial
2) Tested positive for SARS-CoV-2 in the 14 days before the trial
3) Travel outside of Canada in the 14 days before the trial
4) Had signs or symptoms of SARS-CoV-2, as identified on the screening form (see below), on the study days.

COVID-19 Screening Tool

(Please complete prior to coming to school. Completed forms must be brought to school in order to participate in the study. Complete one form each day of the study):

Date: ______________________________

Students's Name: ______________________________(please print)

Grade: ______________________________

© 2022 Science M et al. *JAMA Pediatrics*.

If the answer is "yes" to any of the questions in the chart below, contact XXX prior to bringing your child to the study location	YES	NO
Have you or anyone in your household returned from travel outside of Canada within the last 14 days?		
Have you or anyone in your household had contact with anyone with an acute respiratory illness and has returned from travel within the last 14 days?		
Have you or anyone in your household had contact with anyone who has COVID-19 symptoms (see list below) or who has tested positive for COVID-19, without wearing appropriate personal protective equipment?		
Do you or anyone in your household have the following new or worsening symptoms: • Fever (> 37.8 °C)		
• Cough		
• Sore throat		
• Shortness of breath		
• Difficulty swallowing		
• Decrease or loss of taste or smell		
• Any nausea, vomiting, diarrhea, or stomach pain		
• Runny nose or nasal congestion (in the absence of underlying reasons such as chronic seasonal allergies, chronic nasal drip, etc.)		
• Unexplained tiredness or sore muscles		
• Chills		
• Headache		
• Conjunctivitis (pink eye)		
Have you taken any fever reducing medications (e.g. Tylenol®/Tempra® (acetaminophen), Advil®, /Motrin® (ibuprofen), etc.) in the past 24h?		
I verify that all answers above reflect accuracy of current health conditions. I understand that falsifying any answers could result in putting many others at risk for COVID-19.		

Trial Procedures and Curriculum

Instructions for Teachers

© 2022 Science M et al. *JAMA Pediatrics*.

Teachers had a training day prior to the trial to review the expectations, plan and prepare materials. They were guided through the following agenda and provided time to set up their classrooms with proper distancing between desks and student materials. They also had time to co-plan with their grade partner to promote a consistent curriculum between the mask and control classes.

Agenda - Teacher Prep:

INTRO - Welcome & Thank You

1. Sick Kids Tracking and Coding
2. AM Entry Routine, Entrance/exit enforcement, Fire Exits
3. Outside spaces for recess and lunch
4. Lunch, snacks and drinks
5. Lunch supervision
6. Mask requirements & storage
7. Washrooms
8. Technology, Photocopier (including social media)
9. Support for teacher breaks or student issues
10. Grade Teams & Co planning
11. Schedule / Timetable
12. O Canada and land acknowledgement
13. QUESTIONS, CONCERNS, DISCUSSIONS

Teachers were provided a schedule for the two days. Individual lesson planning for the days was the responsibility of each teacher (no lesson plans were provided). Team planning was encouraged between the same grades to ensure a consistent daily program delivery between the mask and control classes. Teachers were encouraged to plan as ‘normal’ a school day experience as possible to accurately reflect what happens in the classroom.

The school day included language, mathematics, social studies, and the arts in a schedule that adheres to the Ministry of Education’s Protected Time for Daily Math Instruction PPM 160. Resources around mindfulness, wellness and resiliency were provided to teachers.

Teachers were informed of the deviation from the Education Act regarding 40 minutes of uninterrupted lunch and prep time allotments for the two days due to the nature of the study and the need to provide separate safe spaces for lunch, outdoor play, and student supervision.

© 2022 Science M et al. *JAMA Pediatrics*.

Response scripts and recommendations around hand hygiene and mask wearing were provided. Hand hygiene was scheduled up to 7 times in the day around transitions in and out of the classroom, with additional hand washing as needed. All teachers received this information to ensure that consistent protocols were shared with students regarding COVID-19.

All staff and students had a negative COVID-19 test prior to the start of the study. Each day a daily paper-based health screening was completed by all staff and students.
Teachers for students Grade 8 and younger were responsible for the transfer of care of students from parent/caregiver to teacher and back again at the end of the day. Students in Grades 9-12 had the option to travel to and from school on their own with parental/caregiver permission.

Three additional teachers were on site (two at Bishop Strachan School, one at Upper Canada College) during the study to provide classroom coverage as needed, and support for teachers and students.

Instructions for Students

Students were assigned to classrooms across two sites:

- K-4 at School 1
- 5-12 at School 2

Entry and dismissal times were staggered by grade and class, with mask and control classes not entering the school or exiting simultaneously.

1. School 2. Grade 9-12 entered through the back entrance; Grade 5-8 from the front entrance. The floors were marked into sections; each class had a section assigned where they received their coded number sticker.
2. School 1. 1-4 in the back field. Each class had a designated space to line up where they received their coded number sticker. Everyone entered/exited through the same gate.

© 2022 Science M et al. *JAMA Pediatrics*.

3. Kindergarten students met in their dedicated play yard with students staying on the half of the yard where their entry door is located where they received their coded number sticker.

At both schools, attendance was completed outside, prior to entry into the school. Once all students were present, the independent statistician was contacted to randomize students for GloGerm™ application.

At School 1, as each class entered through the main doors, they approached the desk for GloGerm™ application based on the randomization. The photo booth was beside the desk and students moved from one station to the next before waiting along the back wall for the class to finish.

At School 2, as each class entered through the main doors, they approached the table for GloGerm™ application based on the randomization. The photo booth was in the central lobby and students moved from one station to the next before proceeding to their classroom.

Lunch and recess times were staggered by grade and cohort as outlined in the Mask Study 2-Day Timetable (below) so that mask and control classes were not using common spaces at the same time.

Students had mask breaks during the day as needed, during snack and lunch breaks, and at recess. Signage was posted at student eye level in the halls and at measured distances on the floor.

Sample Timetable for Study Days: Kindergarten

Time	Day 1	Day 2
8:35-8:50<br>15 minutes + hand washing	Entry routine	Entry routine
8:50-9:30<br>40 minutes + hand washing	Outdoor play	Outdoor play
9:30-10:10	Discussion – Creating community	Discussion – Creating community

© 2022 Science M et al. *JAMA Pediatrics*.

40 minutes	• O Canada (listening) & Land Acknowledgement<br>• Introduce 2-day agenda<br>• Check-in<br>• Establish collaborative norms, safe space, etc.<br>• Wellness activity/discussion	• O Canada (listening) & Land Acknowledgement<br>• Review 2-day agenda<br>• Check-in<br>• Review collaborative norms (posted on wall)<br>• Wellness activity/discussion
10:10-11:30<br>80 minutes	Inquiry centres	Inquiry centres
11:30-12:20<br>50 minutes + hand washing	Lunch in room	Lunch in room
12:20-1:00<br>40 minutes<br>+ hand washing (pre and post)	Outdoor play	Outdoor play
1:00-2:20<br>80 minutes	Inquiry centres	Inquiry centres
2:20-2:40<br>20 minutes	Discussion – End of day	Discussion – End of day
2:40-2:55<br>15 minutes + hand washing	Dismissal routine	Dismissal routine

© 2022 Science M et al. *JAMA Pediatrics*.

Sample Timetable for Study Days: Grade 1 – 4

Time	Day 1	Day 2
8:35-8:50<br>15 minutes + hand washing	Entry routine	Entry routine
8:50-10:10<br>80 minutes	Discussion – Creating community<br>• O Canada (listening) & Land Acknowledgement<br>• Introduce 2-day agenda<br>• Check-in<br>• Establish collaborative norms, safe space, etc.<br>• Wellness activity/discussion	Discussion – Creating community<br>• O Canada (listening) & Land Acknowledgement<br>• Review 2-day agenda<br>• Check-in<br>• Review collaborative norms (posted on wall)<br>• Wellness activity/discussion
10:10-10:20<br>10 minutes<br>+ hand washing (pre and post)	Outdoor recess 1-6 / Transition Time 7-8	Outdoor recess 1-6 / Transition Time 7-8
10:20-11:30<br>70 minutes	Language	Language
11:30-11:55<br>25 minutes + hand washing	Lunch outdoor recess	Lunch outdoor recess
11:55-12:20<br>25 minutes + hand washing	Lunch in room	Lunch in room
12:20-1:40<br>80 minutes	Math	Math
1:40-1:50<br>10 minutes<br>+ hand washing (pre and post)	Recess / Transition time	Recess / Transition time
1:50-2:30<br>40 minutes	Social studies / Science / Arts	Social studies / Science / Arts
2:30-3:00<br>30 minutes	Discussion – End of day	Discussion – End of day
3:00-3:15<br>15 minutes + hand washing	Dismissal routine	Dismissal routine

© 2022 Science M et al. *JAMA Pediatrics*.

Sample Timetable for Study Days: Grade 5 – 8

Time	Day 1	Day 2
8:35-8:50<br>15 minutes + hand washing	Entry routine	Entry routine
8:50-10:10<br>80 minutes	Discussion – Creating community<br>• O Canada (listening) & Land Acknowledgement<br>• Introduce 2-day agenda<br>• Check-in<br>• Establish collaborative norms, safe space, etc.<br>• Wellness activity/discussion	Discussion – Creating community<br>• O Canada (listening) & Land Acknowledgement<br>• Review 2-day agenda<br>• Check-in<br>• Review collaborative norms (posted on wall)<br>• Wellness activity/discussion
10:10-10:20<br>10 minutes<br>+ hand washing (pre and post)	Recess / Transition time	Recess / Transition time
10:20-11:30<br>70 minutes	Language	Language
11:30-11:55<br>25 minutes + hand washing	Lunch outdoor recess	Lunch outdoor recess
11:55-12:20<br>25 minutes + hand washing	Lunch in room	Lunch in room
12:20-1:40<br>80 minutes	Math	Math
1:40-1:50<br>10 minutes<br>+ hand washing (pre and post)	Recess / Transition time	Recess / Transition time
1:50-2:30<br>40 minutes	Social studies / Science / Arts	Social studies / Science / Arts
2:30-3:00<br>30 minutes	Discussion – End of day	Discussion – End of day
3:00-3:15<br>15 minutes + hand washing	Dismissal routine	Dismissal routine

© 2022 Science M et al. *JAMA Pediatrics*.

Sample Timetable for Study Days: Grade 9 – 12

Time	Day 1	Day 2
8:35-8:50<br>15 minutes + hand washing	Entry routine	Entry routine
8:50-9:30<br>40 minutes	Discussion – Creating community<br>• O Canada (listening) & Land Acknowledgement<br>• Introduce 2-day agenda<br>• Check-in<br>• Establish collaborative norms, safe space, etc.<br>• Wellness activity/discussion	Discussion – Creating community<br>• O Canada (listening) & Land Acknowledgement<br>• Review 2-day agenda<br>• Check-in<br>• Review collaborative norms (posted on wall)<br>• Wellness activity/discussion
9:30-11:22<br>112 minutes	Course A	Course A
11:22-12:05<br>43 minutes<br>+ hand washing (pre and post)	Lunch in room	Lunch in room
12:05-1:57<br>112 minutes	Course B	Course B
1:57-2:35<br>36 minutes	Discussion, Survey – End of day	Discussion, Survey – End of day
2:35-2:45<br>10 minutes + hand washing	Dismissal routine	Dismissal routine

© 2022 Science M et al. *JAMA Pediatrics*.

Outcome Classification

Hand-to-face-contacts were classified for each hand as contact with the following parts of the head and face.

1) mouth and/or nose
2) eyes
3) glasses
4) other non-mucosal part of the face – defined as touching the chin, ears, cheek, forehead
5) central mask – defined as touching the mask over mouth or nose
6) peripheral mask – defined as touching the side of mask or ear loops
7) removing mask – defined as removing both ear loops
8) putting on mask – defined as putting on mask after both ear loops had been removed

Closed-circuit television video recording and storage

A secured closed-circuit television local area network was designed and developed to record and store video data from each classroom. In each classroom four closed circuit television cameras were mounted in each corner providing a wide-angle recording covering the entire classroom including entrances and windows. Video data composed of full color frames of 2688x1520 pixel resolution recorded at 30 frames per second. The videos were recorded using infrared fixed mini dome network cameras (Hik Vision, Hangzhou, China) which were connected to a data video recorder (DVR) (Hik Vision, Hik Vision, Hangzhou China) using CAT5 cables. Access to the network was password controlled via a monitoring station setup in a secured office. Data was stored on the DVR during recording and transferred to the institution's Microsoft Stream (Microsoft, Redmond, CA) account for storage and streaming access by the assigned video coders. The Microsoft Stream service is a cloud-based video stream service that allows for simultaneous access to videos using enterprise assigned usernames and passwords. The setup for each classrooms CCTV cameras is depicted in eFigure 5. For video playback, coders were able to freeze frames, rewind and use slow motion to review each frame of the videos. All four camera angles were available for review.

© 2022 Science M et al. *JAMA Pediatrics*.

Training and Analysis of Video Data by primary and secondary coders

Coders received training by a single trainer (MCK) on how to code hand-to-face-contacts using archive videos and underwent an assessment to ensure consistency of coding of at least 10 minutes of video footage in comparison with the trainer. If there was consistent coding of the primary outcome between MCK and the coder during this assessment, the coder would proceed with their assigned coding of one grade range, including the mask and the control classroom. If there was inconsistency in the primary outcome coding, additional assessments were required until there was consistency.

Due to public health measures and social distancing, classes commenced in a staggered manner. Video from each class was reviewed by MCK to identify a 60-minute period in the morning and a second 60-minute period after lunch break where students were present in the classroom and doing classroom activities. Each 60-minute period did not include breaks taken in the classroom. Following, training, a primary coder was assigned to review the 60-minute segment of video and code using the standardized data collection tool. To verify the primary coder data, a five-minute segment was randomly selected from each of the morning and afternoon 60-minute segments for a total of 10 minutes. A second coder was assigned to code these 10-minute segments. Primary coders and secondary coders did not have access to each other's coding. Coding was done between May 3, 2021, and September 3, 2021. The data were collected using Excel 365 (Microsoft, Redmond, CA).

Status of Secondary Outcome Reporting

The following section summarizes the secondary outcomes that were planned in the protocol and submitted to ClinicalTrials.gov (NCT04531254). We have summarized the status of reporting and rationale for not reporting, where applicable:

1) Total number of hand-to-mucus membrane contacts per participant per hour – reported in Table 2.
2) Total number of hand-to-non-mucus membrane contacts per participant per hour – reported in Table 2.

© 2022 Science M et al. *JAMA Pediatrics*.

3) Total number of instances where participants are within 1 and 2 meters of each other – ***not reported*** and not available. In the original protocol, we had planned to have distancing monitors worn by students to flag when within 1 and 2 meters. Unfortunately, these monitors were not available at the time of the trial and the outcome could not be accurately assessed on video review.
4) Total number of GloGerm™ transfers to another person – reported below.
5) Total number of GloGerm™ transfers to teacher – reported below.
6) Total number of GloGerm™ transfers to a surface – ***not reported*** and not available. Unfortunately, given the lighting in the classrooms at the time of the trial, it was not possible to photograph surfaces to assess for GloGerm™ transfer.
7) Total number of instances of hand holding per participant per hour – reported in Table 2.
8) Total number of touches to another person per participant per hour – ***not reported*** and not available. It was technically challenging to assess student to student contact when students were in groups, and it was decided not to proceed with collecting this outcome given the time it would take to accurately capture this data. Priority was given to accurately assessing the primary outcome of hand-to-face contacts.
9) Total number of hand hygiene actions per participant per hour – ***not reported*** and not available. As the cameras were placed in the classrooms and not available in the washrooms, this outcome was not able to be captured.
10) Total number of mask removals per participant per hour in the mask group – reported in Table 2.
11) Teacher and student concerns measured using study-specific post-simulation questionnaire and semi-structured interviews – results are available and reported in separate sub-studies.[1,2]
12) Human factors influencing behaviours – ***not reported*** and data not currently available. The Co-investigators with expertise in human factors work are currently in the planning phase for a sub-study to re-code the video footage to identify activity types that influence face touching behaviour.

© 2022 Science M et al. *JAMA Pediatrics*.

Protocol Deviations

The original protocol planned for 6 groups (Kindergarten, Gr 1-2, Gr 3-4, Gr 5-6, Gr 7-8, Gr 9-12) because we anticipated having difficulty recruiting for high school (Grades 9-12). However, we had no difficulty recruiting and decided to split high school into grades 9-10 and 11-12 for curriculum purposes, resulting in 7 age groups in the study (Kindergarten, Gr 1-2, Gr 3-4, Gr 5-6, Gr 7-8, Gr 9-10, Gr 11-12).

GloGerm Procedures

On day 1 and again on day 2 of the simulation, students in both mask and control classes were centrally randomized in a 1:4 ratio to either a biotracer (GloGerm) or placebo to represent infectious droplets. Students, teachers, and video assessors were blinded to the allocation to biotracer or placebo.

The biotracer was applied upon entry into the school. All students performed hand hygiene with alcohol-based hand rub (hand sanitizer) and then had either GloGerm or placebo applied to their hands with instructions to rub their hands as they did with hand sanitizer.

Photo Processing:

Photos of each participant's hands and face were taken individually at the beginning and end of the day. All photos were taken using smartphones (SM-A515W, Samsung, South Korea) with the built-in camera app and flash off. Photos of the hands were taken by asking participants to insert them into a box placed on a table. The frame of the box was created with T-slot aluminum bars (80/20 Inc., US) and a fluorescent UV light fixature was mounted on top. Black translucent sheets covered the entire box such that photos taken within showed good contrast between areas with and without GloGerm™. Photos of the face were taken by asking participants to enter an enclosed space created in the same way as the box for the hands. Photos were initially stored on the smartphones and then promptly moved to a cloud storage service (OneDrive, Microsoft, US) for later processing.

The photos of the participants were taken at the following time periods: Start of simulated school day and End of simulated school day. The lighting condition of the room was lit with natural

© 2022 Science M et al. *JAMA Pediatrics*.

external light where an enclosed space draped with black translucent sheets were used to block out external lighting. The draped enclosed space measured approximately 4ft x 4ft x 3ft.

Each of the photos was viewed and analyzed with Windows 10 Photos App (Microsoft, US). Two reviewers examined a photo with only the GloGerm™ gel under the UV light source to provide a baseline of how it would appear. The presence of GloGerm™ gel appeared in the photo as a distinct bright white area. All photos of the participants' hands and faces were manually reviewed to determine if a similar white area appeared. In some cases, portions of the photo were enlarged for a more detailed view. During review of the photos, there were some examples where a bright white area was attributed to clothing or the mask fabric and not GloGerm™.

Glogerm Results

Biotracer contamination was defined as the presence of biotracer on the hands and faces of teachers and of students not allocated to the biotracer, using bioluminescent photography in a dark room at the end of the second school day. Biotracer contamination of students and teachers did not differ significantly between groups.

**Outcome**	**Mask Group**		**Control Group**		**Rate Ratio or Risk Ratio (95% CI)**	**p-value**
	(87 students; 172.6 hours)		(84 students; 168.0 hours)			
	**N of events**	**Rate or Risk**	**N of events**	**Rate or Risk**		
**Student biotracer contamination***	3	4.3%	3	4.5%	0.96 (0.23 to 4.03)	___
**Teacher biotracer contamination$^{\phi}$**	0	0.0%	1	14.3%	0.33 (0.02 to 7.02)	___

* There were 18 students in each group tagged with biotracer at the start of the simulation; we therefore report the number of students among those not tagged with biotracer, who had biotracer at the end of Day 2: 69 students contributing 136.9 hours in the masking group and 66 students contributing 132 hours in the control group.

$^{\phi}$ There were 7 teachers contributing 13.8 hours in the masking group and 7 teachers contributing 14.0 hours in the control group; all were included in the analysis; we used a continuity correction of 0.5 to derive the risk ratio with 95% confidence interval.

© 2022 Science M et al. *JAMA Pediatrics*.

eFigure 1. Closed-circuit Television camera setup for each classroom

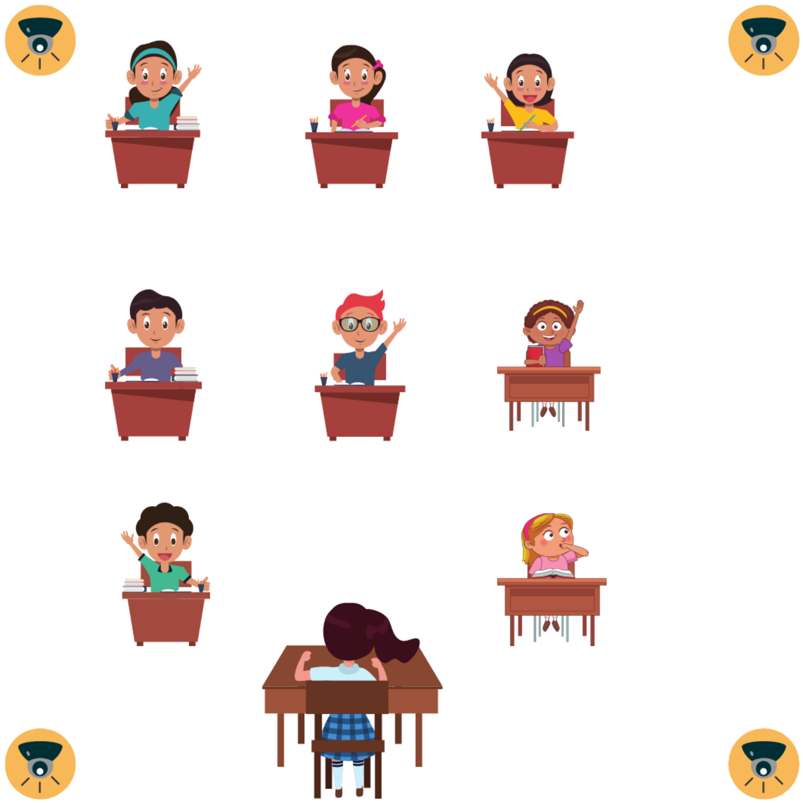

© 2022 Science M et al. *JAMA Pediatrics*.

eFigure 2. Sequential analysis

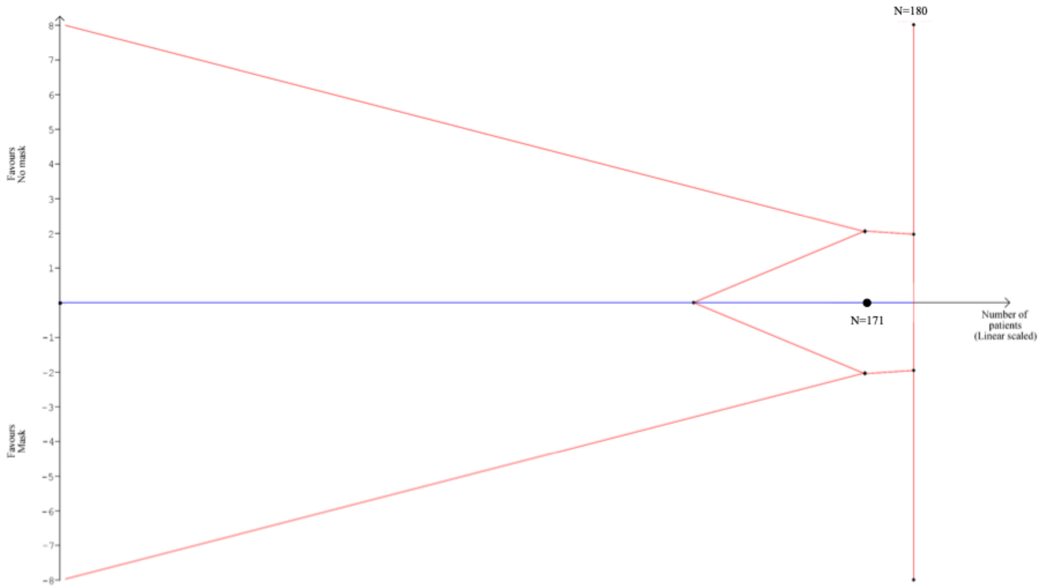

Fixed-effects sequential analysis[3] with outer Lan-DeMets sequential monitoring boundaries[4] and inner equivalence boundaries[5]. The trial was stopped after the pre-planned interim analysis after the first two days of simulation at a sample size of N=171 because the two-sided z of 0.00 (blue line) crossed the boundary of equivalence (inner wedge).

The trial protocol prespecified an adaptive, sequential design. If the first round of simulations reached the information size, or if pre-specified monitoring boundaries for either a difference or for equivalence are crossed after the first round of simulations, the trial would be stopped. If neither the information size was reached nor a boundary crossed, a second round of simulations would be done increasing the sample size as appropriate to reach conditional power of 80% under the alternate hypothesis given the accumulated data. As the equivalence boundary was crossed, we did not do a second round of simulations.

© 2022 Science M et al. *JAMA Pediatrics*.

eFigure 3. CONSORT Diagram for the trial

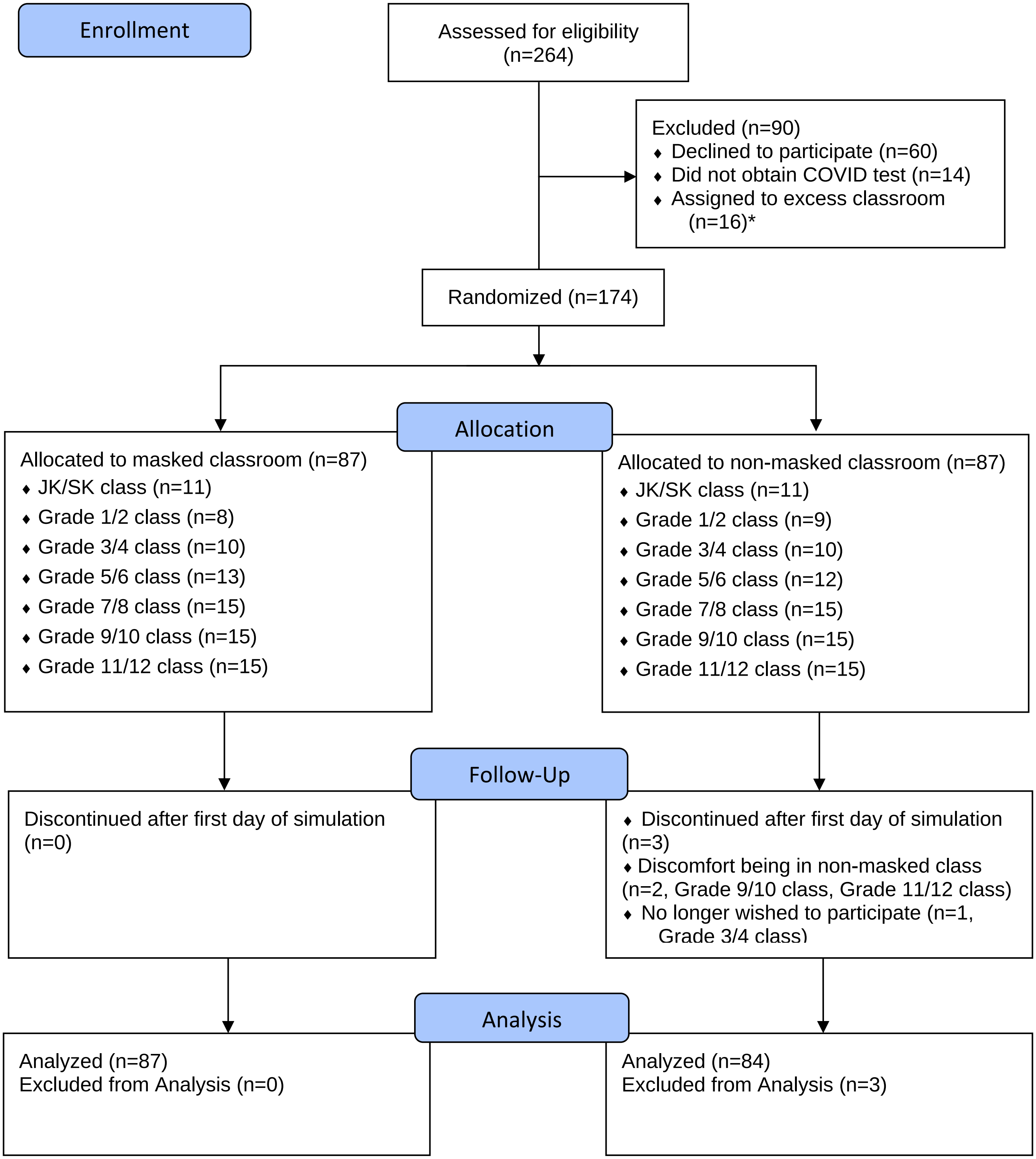

*Class sizes were capped at 15 students as per Ontario Ministry of Education guidelines; students in these grades who were assessed after the maximum class sizes were reached were excluded.

© 2022 Science M et al. *JAMA Pediatrics*.

eFigure 4. Subgroup Analyses evaluating the impact of face mask wearing on the hand-to-face contact

Subgroup	Rate (Masked)	Rate (Control)	Rate Ratio (Mask/Control)	P–value
**Sex**				
Male	77.5	77.4	1.00 (0.77 , 1.30)	0.81
Female	92.0	92.3	1.00 (0.77 , 1.29)	
**School type**				
Public	82.8	84.3	0.98 (0.77 , 1.26)	0.81
Private	83.8	86.0	0.97 (0.76 , 1.25)	
**Ethnicity**				
White	89.1	87.9	1.01 (0.77 , 1.33)	0.34
Asian, Black, Other	80.5	81.9	0.98 (0.75 , 1.29)	
**Age group**				
Gr 1–4	68.2	77.0	0.89 (0.64 , 1.23)	0.46
Gr 5–12	99.5	90.5	1.10 (0.83 , 1.46)	

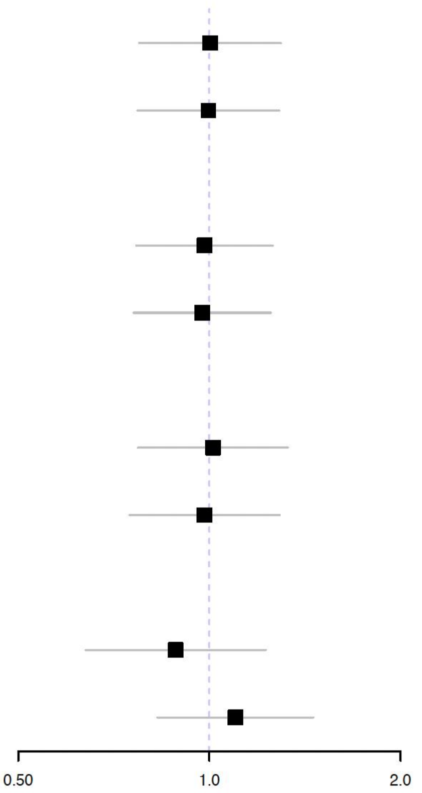

*Marginal estimates from the model

© 2022 Science M et al. *JAMA Pediatrics*.

eFigure 5. Bland-Altman plots depicting agreement between pairs of coders on the study primary outcome

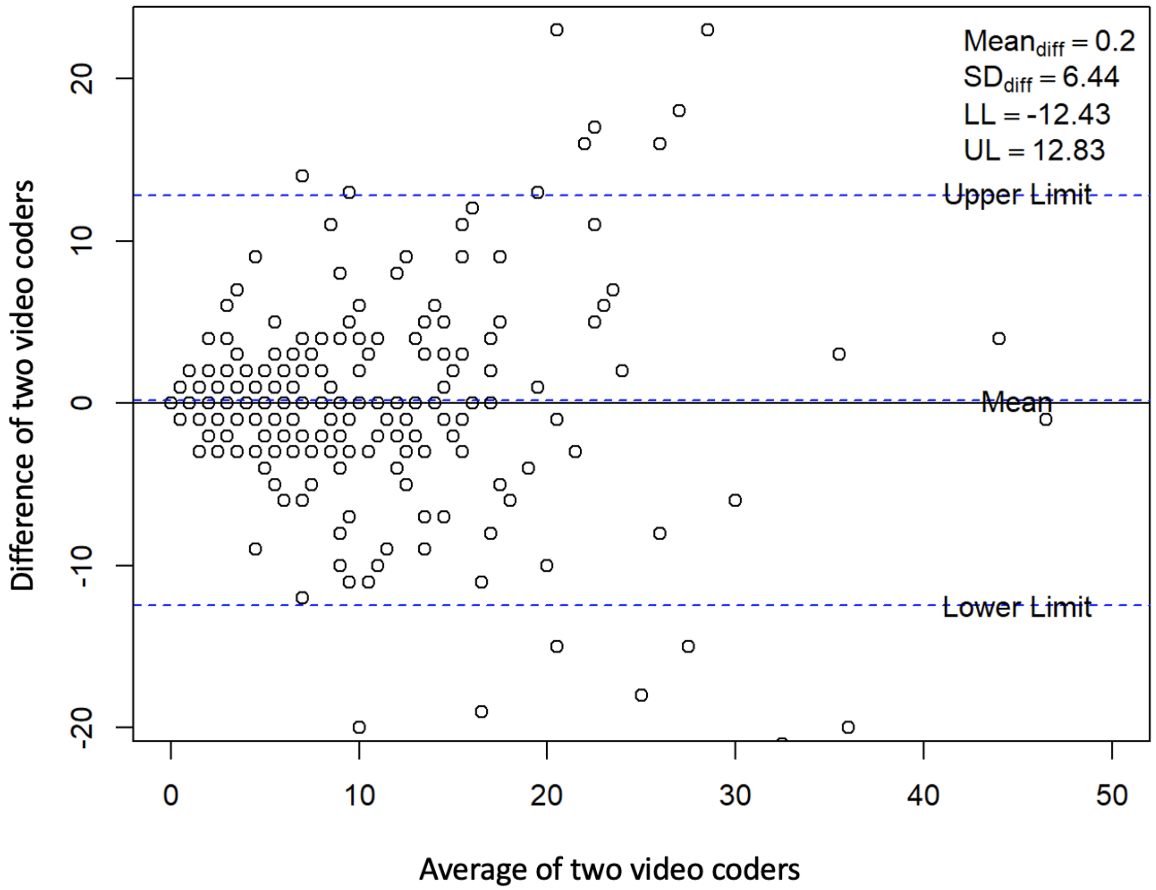

© 2022 Science M et al. *JAMA Pediatrics*.

eTable 1. Intraclass Correlation Coefficients for pairs of coders

	Intraclass Correlation Coefficients	95% Confidence Intervals
Primary outcome	0.72	0.67 to 0.77
Mucosal touches	0.83	0.79 to 0.86
Non-mucosal touches	0.72	0.67 to 0.77
Mask touches	0.80	0.76 to 0.83

© 2022 Science M et al. *JAMA Pediatrics*.

eTable 2. Sensitivity Analysis

Outcome	Mask Group		Control Group		Rate Ratio (95% CI)	p-value
	(83 students; 165.1 hours)		(68 students; 136.0 hours)			
	N of events	Rate per hour	N of events	Rate per hour		
**Hand-to-face contacts**	14901	90.3	11861	87.2	0.95 (0.75, 1.20)	0.66
**Hand-to-mucosa contacts**	625	3.9	3897	28.7	0.10 (0.06 to 0.18)	___
**Hand-to-non-mucosa contacts**	14147	85.7	7697	56.6	1.36 (1.05 to 1.75)	___
**Hand-to-mask contacts***	10251	62.1	1046	7.7	18.7 (3.01 to 116.66)	___
**Hand-to-other face contacts***	3564	21.6	6491	47.7	0.40 (0.28 to 0.55)	___

* Post Hoc Analyses

© 2022 Science M et al. *JAMA Pediatrics*.

eTable 3. Breakdown of number of face or mask contact by code classification

Event Type	**Overall** Median number of contacts per hour (min, max)	**Mask Group** Median number of contact per hour (min, max)	**Control Group** Median number of contacts per hour (min, max)
**Mouth and/or Nose**	6.5 (0.0, 106.5)	1.0 (0.0, 49.6)	20.5 (0.5, 106.5)
**Eye**	0.0 (0.0, 12.5)	0.0 (0.0, 4.0)	0.0 (0.0, 12.5)
**Glasses**	0.0 (0.0, 106.5)	0.0 (0.0, 106.5)	0.0 (0.0, 23.0)
**Other – non-mucosal face**	27.0 (0.5, 140.5)	15.0 (0.5, 106.0)	37.5 (21.9, 58.6)
**Central Mask**	8.5 (0.0, 118.0)	22.0 (0.0, 118.0)	1.3 (0.0, 102.5)
**Peripheral Mask**	7.5 (0.0, 116.5)	20.5 (1.5, 116.5)	1.0 (.0, 59.5)

© 2022 Science M et al. *JAMA Pediatrics*.

References

1. Lin J, Pol SJ, Korczak DJ, et al. Impact of COVID-19 Public Health Protocols on Teachers Instructing Children and Adolescents During an In-Person Simulation. J Can Acad Child Adolesc Psychiatry 2022;31(2):52-63. (https://www.ncbi.nlm.nih.gov/pubmed/35614958).
2. Coelho SG, Segovia A, Anthony SJ, et al. Return to school and mask-wearing in class during the COVID-19 pandemic: Student perspectives from a school simulation study. Paediatr Child Health 2022;27(Suppl 1):S15-S21. DOI: 10.1093/pch/pxab102.
3. Wetterslev J, Jakobsen JC, Gluud C. Trial Sequential Analysis in systematic reviews with meta-analysis. BMC Med Res Methodol 2017;17(1):39. DOI: 10.1186/s12874-017-0315-7.
4. Lan K, DeMets D. Discrete sequential boundaries for clinical trials. . Biometrika 1983;70:659-63.
5. Jennison C, Turnbull B. Group sequential methods with applications to clinical trials. CRC Press 1999.

© 2022 Science M et al. *JAMA Pediatrics*.
