# Supplementary material for: Effect of Wearing a Face Mask on Hand-to-Face Contact by Children in a Simulated School Environment: The Back-to-School COVID-19 Simulation Randomized Clinical Trial
Source: JAMA Pediatr. 2022 Oct 24;176(12):1169–75. doi: 10.1001/jamapediatrics.2022.3833 (PMC9593317; doi:10.1001/jamapediatrics.2022.3833)
Supplement: Supplement 3. — Summary of Protocol Changes [file jamapediatr-e223833-s003.pdf]

Protocol: Understanding the Impact of Public Health Measures Planned for School Reopening during COVID-19 using Simulation Exercises (Back to School Study)
Version: 2.0, 22-Sep-2020

<u>Summary of Changes</u>

An amendment has been drafted to the Back to School study protocol. The following is a summary of key changes to the previous version of the protocol (1.0, August 6, 2020) including the rationale for each change. Formatting changes and minor administrative changes (typos, grammatical changes, minor clarifications in wording) are not included in this table.

Original Text	Modified Text	Rationale
[Text addition]	A fourth REDCap questionnaire will be distributed to the teachers to collect demographic information (Data Collection Form 4 – Teacher Demographic)	We wished to be able to provide demographic information for our teachers (which was subsequently reported in a teacher qualitative substudy manuscript)

Protocol: Understanding the Impact of Public Health Measures Planned for School Reopening during COVID-19 using Simulation Exercises (Back to School Study)
Version: 3.0, 06-Feb-2021

Summary of Changes

An amendment has been drafted to the Back to School study protocol. The following is a summary of key changes to the previous version of the protocol (2.0, September 22, 2020) including the rationale for each change. Formatting changes and minor administrative changes (typos, grammatical changes, minor clarifications in wording) are not included in this table.

Original Text	Modified Text	Rationale
The **primary outcome** will be the number of hand-to-face contacts per hour per student.	The **primary outcome** will be the number of hand-to-face contacts per hour per student on day 2	We wished to clarify that measurement was only intended to take place on Day 2
Self-reported experiences of students and teachers in a qualitative substudy.	Self-reported experiences of students and teachers in a qualitative substudy (e.g., anxiety, mental health)	We wished to clarify what type of information was being sought via the self-reported experiences.
We will use a mixed linear regression model adjusted for age and sex with a random intercept for class to analyse the primary outcome. 95% confidence intervals will be bootstrapped.	We will use a mixed linear regression model adjusted for age and sex with a random intercept for class to analyse the primary outcome. 95% confidence intervals will be bootstrapped. Subgroup analyses planned *a priori* included sex, ethnicity, school type (private vs. public), no mask recommendation (JK – Gr 4 vs. Gr 5 – 12)	Upon finalizing the manuscript it came to our attention that we had not indicated in the protocol that our results would be subject to subgroup analyses; however, this was always our intention and so the *a priori* subgroups were specified. This protocol change was done prior to outcome ascertainment.
