# Supplementary material for: Effect of Wearing a Face Mask on Hand-to-Face Contact by Children in a Simulated School Environment: The Back-to-School COVID-19 Simulation Randomized Clinical Trial
Source: JAMA Pediatr. 2022 Oct 24;176(12):1169–75. doi: 10.1001/jamapediatrics.2022.3833 (PMC9593317; doi:10.1001/jamapediatrics.2022.3833)
Supplement: Supplement 4. — Final Protocol [file jamapediatr-e223833-s004.pdf]

Understanding the Impact of Public Health Measures Planned for School Reopening during COVID-19 using Simulation Exercises

Principal Investigators:
Michelle Science, Infectious Disease Physician, The Hospital for Sick Children
Clyde Matava, Anesthesia, Department of Anesthesia and Pain Medicine, The Hospital for Sick Children

Co-Investigators:

Ari Bitnun – The Hospital for Sick Children (Infectious Diseases)

- Upton Allen – The Hospital for Sick Children (Infectious Diseases)
- Shaun Morris – The Hospital for Sick Children (Infectious Diseases)
- Ronni Cohn – The Hospital for Sick Children (General Pediatrics)
- Jeremy Friedman – The Hospital for Sick Children (General Pediatrics)
- Eyal Cohen – The Hospital for Sick Children (General Pediatrics)
- Catherine Birken – The Hospital for Sick Children (General Pediatrics)
- Laurie Streitenberger – The Hospital for Sick Children (Infection Prevention and Control)
- Daphne Korczak – The Hospital for Sick Children (Psychiatry)
- Samantha Anthony – The Hospital for Sick Children (Qualitative Research)
- Rachel Solomon – The Hospital for Sick Children (Chief Data Officer)
- Lennox Huang – The Hospital for Sick Children (Critical Care, Simulation)
- Emily Louca – The Hospital for Sick Children (Simulation)
- Alison Dodds – The Hospital for Sick Children (Simulation)
- Sunayna Vuppal – The Hospital for Sick Children (Simulation)
- Stacie Carroll – The Hospital for Sick Children (Teacher)
- Giovanna Panzera – The Hospital for Sick Children (Teacher)
- Jodi Greenwood – The Hospital for Sick Children (Teacher)
- Laura Alexander – The Hospital for Sick Children (Occupational Health and Safety)
- Rulan Parekh – The Hospital for Sick Children (Research Methodology)
- Catherine Walsh – The Hospital Sick Children (Simulation, Human Factors)
- Sloane Freeman – Unity Health (Pediatrics)
- Doug Campbell – Unity Health (Simulation, Human Factors)
- Peter Jüni – St. Michael's Hospital, IHPME (Research Methodology, Statistics)
- Bryan Maguire – The Hospital for Sick Children (Data Analysis, Statistics)
- Derek Stevens – The Hospital for Sick Children (Data Analysis, Statistics)
- Mark Crawford – The Hospital for Sick Children (Research Methodology, Simulation)
- Monica Caldeira – The Hospital for Sick Children (Research Coordinator)
- Allison McGeer – Mount Sinai Hospital (Research Methodology, Infection Prevention and Control)
- Dominik Mertz – McMaster Hospital (Research Methodology, Infection Prevention and Control)
- Sarah Khan – McMaster Children's Hospital (Pediatric Infection Prevention and Control)
- Maureen Cividino – Public Health Ontario (Occupational Health and Safety)
- Jessica Hopkins – Public Health Ontario (Public Health)
- Laura Bourns – Public Health Ontario (Public Health)
- Kevin Schwartz – Unity Health (Pediatric Infectious Diseases)
- Vinita Dubey – Toronto Public Health (Public Health)

1.0 INTRODUCTION

1.1 Overview: The American Academy of Pediatrics[1] and the Canadian Pediatric Society[2] have issued statements emphasizing the importance of children returning to school for broader child health. The Ministry of Education has released guidance for return to school during the COVID-19 pandemic which includes recommended health and safety measures.[3] However, there are limited details provided around the implementation of these measures.

1.2 COVID-19 in children: Multiple reports from around the world indicate that children account for less than 5-10% of SARS-CoV-2 infections.[4-6] In Canada, of 104,370 COVID-19 cases reported as of July 4th 2020, 7,470 (7.16%) were in children aged 0-19 years.[7] While this may, at least in part, be related to testing practices and early school closure, evidence is mounting that children may be less susceptible to SARS-CoV-2 infection and less likely to transmit the virus to others.[8-10] There is also strong evidence that the majority of children who become infected with SARS-CoV-2 are either asymptomatic or have only mild symptoms, such as cough, fever, and sore throat.[4,5,11-13] While serious disease requiring hospitalization is known in children, including multisystem inflammatory syndrome in children (MIS-C), this is relatively rare and is generally treatable.[14-16] Severe disease requiring intensive care admission occurs in a small minority of paediatric cases, particularly among those with certain underlying medical conditions, but the clinical course is much less severe than in adults and deaths are uncommon.[4,6,17,18]

1.3 Transmission risk from children and in schools: While the concerns around infection and infectious complications in children appear to be minimal, it is important to consider the potential role children may play in transmission (especially to vulnerable individuals both inside and outside of the classroom) and disease propagation. Children are considered to be efficient transmitters of influenza and other respiratory virus infections and this was one of the rationales for early school closures. However, data from multiple countries is emerging that children do not play a predominant role in the transmission of SARS-CoV-2.[8,9,19-21] This is further supported by the evidence to date that schools do not appear to have played a significant role in transmission[22] and even when cases have been identified in schools, contact tracing and testing have not identified a large number of secondary cases.[23-26] Furthermore, several countries have reopened schools without demonstrating a significant increase in cases.[23-27]

1.4 Risk mitigation strategies: Despite the reassuring evidence cited above, it should be recognized that it will not be possible to remove all risk of infection and disease now that SARS-CoV-2 is well established in many communities. Mitigation of risk, while easing restrictions, will be needed for the foreseeable future to balance health with social and economic goals. The mitigation strategies implemented for school reopening have varied from country to country, ranging from strict enforcement of physical distancing and masking, to no masking and emphasis on cohorting to allow for close interactions. While outbreaks have been reported in schools in some countries (e.g. Israel[28]), the risk mitigation strategies appear to have been largely successful in the majority of other countries.[23-26] The Ministry of Education has released guidance for return to school during the COVID-19 pandemic which includes recommended health and safety measures.[3] However, there remains controversy around how several measures will be implemented upon return to school.

1.5 Face masks/coverings in children: In particular, the use of face masks/coverings in children of various ages has been suggested as an important element to reducing SARS-CoV-2 (COVID-19) transmission in the school setting.[29] In contrast, others have raised concerns that this may lead to an increased risk of infection due to increased touching of the masks and face by children and will be difficult to enforce, especially in younger children.[1] The Province of Ontario has not mandated the use of facemasks/coverings in indoor spaces which is contrary to many local bylaws, although schools are excluded from the bylaws.[30]

Unfortunately, there is limited data on masking in children on which to make evidence informed decisions. Some studies have evaluated the habitual and voluntary behaviours such as face mask-wearing and hand hygiene in children and adolescents. Wong et al (2005)[31], in a telephone survey of adolescents following SARS in Hong Kong, reported that only 54.8% of 230 respondents reported practicing all recommended behaviours that included face mask wearing and hand hygiene. While the study does not identify whether these adolescents practiced these behaviours in school or at home, the presence of environmental cues and a high perceived health threat were factors associated with increased compliance. In a questionnaire study among all 13,217 elementary school children in Matsumoto City in Japan, the self-reported use of face masks was found to offer higher protection from self-reported influenza diagnosis by a physician (2014/15 season) in older children (Grade 4-6) than younger one (Grade 1-3).[32] The ability of older children to control their health-protective and infection control measures and activities such as face mask wearing and handwashing were proposed as possible reasons for the protection conferred in the older age group. A randomised cluster trial of households during the 2009/2010 and 2010/11 influenza seasons in Germany compared masking, masking and hand hygiene (HH) or control in 84 households[33]. There was no significant effect from either intervention in the primary analysis. There was a potential effect observed in the subgroup that implemented masking + HH within 36 hours of symptom onset of the index case (adjusted odds ratio (OR) 0.16, 95% CI, 0.03-0.92). There have been no prospective studies evaluating the impact of face mask wearing vs. no face mask in the school environment.

1.5 Relevance: It is critical to gain a better understanding of the benefits and risks associated with the proposed health and safety measures and how teachers and students can be protected. Real-world simulation exercises provide an excellent opportunity to gather this information in a smaller setting and to understand implementation considerations prior to broader implementation of any of these health and safety measures for the start of the school year.

2.0 OBJECTIVES

The primary objective of this study is to evaluate the impact of the use of face masks/coverings, across various children's age groups, on hand-to-face contact among students in a simulated class environment. Secondary objectives include:

- To evaluate the impact, across various children's age groups, of the use of face masks/coverings compared to no masks at school on the spread of a safe biological indicator (fluorescein or Glo Germ) from a group of children within a classroom to 1) the hands/face of classmates or the teacher within the same classroom and 2) to other surfaces in the classroom.
- To evaluate the impact, across various children's age groups, of wearing facemasks versus no facemasks in a class at school on 1) other personal behaviours (i.e. mask removal, hand-to-mucous membrane contact), 2) person to person interactions and 3) teacher and child experiences based on their days in class.
- To evaluate the use of face shields compared to face masks/coverings worn by teachers as personal protective equipment in terms of 1) effectiveness as source control (using monochromatic camera and dark room), 2) teacher opinions including preference (compared to face mask), ability to communicate, comfort, and 3) student opinions, including comfort with the teacher, ability to learn, distractibility, opinion.
- To simulate the environment upon return to school with the purpose of 1) identifying teacher and student concerns with a focus on physical distancing and hand hygiene, 2) determining the amount of time children are within both 1 meter and 2 meters of other students inside and outside of the classroom environment and 3) number of hand hygiene events per child per day.

3.0 RESEARCH PLAN:

3.1 Brief Overview: This will be a prospective study simulating return to school for two full days prior to the start of the school year in September. Volunteer students and teachers from six grade ranges will be recruited to participate in the study. In each grade range, there will be two classes that will participate, one that will be assigned to wear face masks/coverings (experimental group/class) and the other that will not wear any face masks/coverings (control group/class). Students will be randomized to either the masking (experimental) or no masking (control) class in their appropriate grade. Personal behaviours and person-to-person interactions will be recorded using video cameras. In addition, a safe biological indicator will be applied to the hands and either mask or tip of the nose to simulate potential asymptomatic infection in a subset of students. Other students will have water applied so that students are blinded to who has the indicator. Cameras will be used to document how the indicator moves throughout the classroom.

3.2 Study Site: The study will occur at Upper Canada College (UCC) and Bishop Strachan School (BSS) to run a mock curriculum over two days prior to the start of the school year in September.

3.3. Study Population: Grade ranges will include 1) Senior Kindergarten (children who have just completed JK or SK in the 2019-2020 year), 2) Grades 1-2, 3) Grades 3-4, 4) Grades 5-6, 5) Grades 7-8 and 6) high school. A total of 12 teachers and up to 240 students will be recruited. Final class sizes will be in accordance with Ministry of Education and public health guidelines.

3.4 Inclusion and exclusion criteria

To be included in the study, students must have attended school during 2019-2020 school year and provide informed consent (student in Grade 7 or older) or parental consent (SK – Grade 6) and assent (if appropriate). Students will be excluded if they:

- Require additional resources or support beyond what can be provided by the single class teacher
- have been exposed to an individual that has tested positive for SARS-CoV-2 in the 14 days prior to the simulation
- test positive for COVID-19 in the 14 days prior to the simulation
- have signs or symptoms of COVID-19, as identified on the screening form (See Screening Form) before the simulations
- travel outside Canada in the last 14 days
- known hypersensitivity or allergy to the biological indicator (Fluoroscein dye/GloGerm)

To be included in the study, teachers must be certified teachers and provide informed consent to participate. Teachers will be excluded if they:

- have been exposed to an individual that has tested positive for SARS-CoV-2 in the 14 days before the simulation
- test positive for COVID-19 in the 14 days before the simulation
- have signs or symptoms of COVID-19, as identified on the screening form (See Screening form) before the simulations
- travel outside Canada in the last 14 days
- known hypersensitivity or allergy to the biological indicator (Fluoroscein dye/GloGerm)

3.4.1 Recruitment: Students and teachers will be approached by school principals. Recruitment will be directed first to students and teachers from the GTA and broadened to other areas if additional participants are needed. This communication will direct participants to a website that will contain study information and provide details about how to gain access to webinars that will provide more information. While

detailed information will be provided, this will not replace the full consent process. At a date following the occurrence of the webinars, individuals will be provided the option to indicate their plan to participate in the study via an online form. This form will ask the parent/student to indicate the child's sex, which grade they just completed, in addition to contact information. This online sign up will be followed up with a call (telephone or virtual platform – see below) from the research coordinator to start the consent discussion, finalize decisions around consent and assent (ideally via teleconference) and proceed to the online consent/signature process. Consent will be obtained from students if they are deemed to have capacity to consent (likely Grade 7 and above, but to be confirmed based on capacity assessment). If a student provides informed consent to participate in the study, a parental permission form will also be completed to document their support for the study. For students who do not have capacity to consent, parental consent will be obtained (likely from students Grade 6 and younger). The assent form will be reviewed and assent will be verbal and will be completed at the time of parental consent and documented on the REDCap form. The aim will be for this to occur over a virtual platform with video capability in order for the research coordinator to assess willingness to participate.

- Attempt to obtain consent over Microsoft teams (or other approved platform), but telephone is an alternative if it is not feasible given limitations to technologies
- A capacity assessment and willingness to continue to participate will be confirmed in person prior to doing NP swab testing. This will include two questions 1) do you understand the study and what will be happening if you are involved? 2) are you willing to participate?

Active communication: The local principal will reach out to potential participants via email (See Email Communication and Poster). We will ask them to include details about our study, including the website information and the RC and PI contact information if they are interested.

For teachers, options for recruitment may include emails and communication from leadership. The process will be approved by the school and they will be responsible for determining the teachers that will be invited to participate. This communication will direct teachers to a website that will contain study information and the study coordinator and PI information will be provided so that teachers can indicate their interest in participating independent of the school. Once teachers indicate interest in participation, the SickKids study team will contact the teacher to go through the virtual consent process and online consent signature.

Incentives: Upon completion of the study, students will be provided with volunteer hours and a $50 Amazon gift card. Teachers will be compensated for the two days of teaching ($200/day) and the testing/training days ($100), for a total of $500.00.

3.5 Study Methods

3.5.1: Study cohort: We will recruit up to 240 students and 12 teachers for the simulation. Once recruited, information on both students and teachers will be collected using an online REDCap survey, including name, full date of birth, address and contact information in order to register the participant in the SickKids Epic Research Module to facilitate ordering of a COVID-19 swabs (Data Collection Form 1 – Registration). For students, the school health questionnaire (as per school requirements) will also be completed using the online REDCap survey to document any underlying health conditions, medications, allergies and other special requirements for the safe operation of school (Data Collection Form 2 – Health Form). This information will not be linked to any study data, but will be provided to the school and the student's teacher. A third data collection form will be completed via Redcap by the parent and student around their concerns and views related to public health measures for school (Data Collection Form 3 – Baseline Survey). This form will be de-identified after completion and linked only to the classroom to which the student is assigned. It will need to be linked to the classroom to adjust for any baseline differences between the

experimental and control groups. A fourth REDCap questionnaire will be distributed to the teachers to collect demographic information (Data Collection Form 4 – Teacher Demographic).

3.5.2: Randomization and blinding: Once teachers and students are recruited; students will be assigned to one of two appropriate classes based on the grade they just completed: students assigned to wear masks will be placed in one class; the other class (herein referred to as the 'no mask' group) will be for students who will not be wearing masks (Grades 4 and lower) or wearing masks only when physical distancing cannot be maintained (Grade 5 and up). Central randomization will be done one to two days prior to the simulation using an adequately concealed interactive web-based response system in REDCap (see below), based on computer-generated randomization schedules stratified by age and sex. The allocation will be communicated to parents and students at least half a day before the start of the simulation to ensure appropriate preparation time. Teachers will wear a face shield if within 2 m of students in both experimental and control arm during day 1 (implementation phase), and face masks if within 2 m of students in both experimental and control arm during day 2 when outcome data will be collected. Students and teachers aware that personal behaviours are being recorded, but will be blinded to the primary outcome (hand-to-face contact) and other specific behaviours that are being monitored.

On each of day 1 and day 2 of the simulation, students in both experimental and control arm will be randomized in a 1:4 ratio to either a Bio-tracer versus a placebo Bio-tracer. This corresponds to a frequency of the Bio-tracer of 20%, to represent the upper range of asymptomatic colonization. Students, teachers and video assessors will be blinded to which students have bio-tracer or placebo.

3.5.3: Pre-Simulation: Prior to the simulation, an information package will be distributed to families including information about COVID-19 testing (procedure, location, what happens if the test is positive), logistics for the simulation (arrival and departure times, what to bring) and general safety information (hand hygiene information and facemask, if appropriate based on randomization) (Appendix X - Back to School Package). Testing for COVID-19 will be completed on teachers and students by the SickKids mobile testing unit at the school 24-48 hours before the first day of the simulation to minimize the risk of COVID-19 transmission during the simulation experiment. Students and teachers will not be required to self-isolate following the test, in keeping with current recommendations for asymptomatic testing. A workflow will be instituted onsite to ensure that the testing is completed in a timely manner to avoid crowds. Information will be provided to participants and families about the necessary steps should the swab be positive. The swab results will be used for Toronto Public Health reporting reasons only and will not be linked to study data (positive participants will be excluded from participating – see inclusion and exclusion criteria).

3.5.4 Intervention / Simulation:

Simulation Scenario Setting: The simulation will occur at UCC and BSS in designated classrooms. Policies and procedures will replicate the plans for return to school in September. Hand hygiene (either alcohol-based hand rub or soap and water) will be available and regular cleaning will occur by trained staff throughout the simulation days. Students in the face mask classes will be instructed to bring their own face mask/covering to wear throughout the school day. Face masks/coverings will be available at the school if a student assigned to a masking class forgets or loses their face mask. In order to record the classroom and school activity for review and analysis, a secure and privileged CCTV set-up commonly used in simulation studies will be used on a closed mesh network. Six cameras will be set up in each classroom and additional cameras in common spaces (play-ground, hallways). An observer will be placed in the bathroom to observe hand hygiene, distancing and mask use. Proximity sensors will be worn by each participant and will log each encounter where participants are within 1 m and 2 m of other participants. Both the video recording and proximity sensors will be included in the consent process.

Simulated Curriculum: The curriculum for the school days has been developed by teachers (See Curriculum) to simulate a regular school day. It will include a brief overview of mindfulness strategies to

help coping with any anxiety around return to school or around mask-wearing. There will be no COVID-19 specific material included in the curriculum as this may be triggering for some students and precipitate increased anxiety (recommendation from SickKids Psychiatry). However, teachers will be given resources and training on how to handle questions should they arise (See COVID-19 Q+A for teachers – developed by Infectious Diseases, approve by Public Health). Mental health resources will be available on site on both simulation days (social work, psychology) should the simulations trigger anxiety.

Simulation Day Procedures: Students will arrive at school as outlined in the "Back to school package". Procedures will be in accordance with the school plans for return to school in September (i.e. staggered start times). On arrival, students in each class will congregate in a designated area (i.e. outside, weather permitting). When the class is assembled, a safe invisible biological indicator (Fluorescein) will be applied to the hands and the tip of the nose to a random 3 students in each class to be consistent with the potential for up to 20% asymptomatic infection in children. Other children will have water applied so that teachers and students are blinded to the students with the indicator. The children's hand, face and classroom environment will be photographed 3 times per day as to identify the fluorescent indicator. Video recording will be utilized to record all events with cameras mounted in each classroom and public recess area. Proximity sensors worn by each participant will log non-identifying log data on the number of durations of proximity contact. The students will enter the classroom and the curriculum will proceed as outlined in "simulated curriculum" above. Students will bring their lunches and eat in their assigned classroom. Mental health resources will be available on site should any concerns arise for teachers or students.

For teachers, simulation exercises will be done with face shields to evaluated their effectiveness as source control (Appendix X – Teacher Face Shield Experiment Procedure).

Video Coding: A trained rater will watch the video recorded simulations independently. Initially, key events and actions in the videos (e.g., touching face, removing a mask, activities causing proximity of students) will be time stamped. Subsequently codes will be assigned to key events using the SEIPS framework to identify safety threats and categorize them by theme. The SEIPS model depicts a system as a human-centered with 6 interacting components that influence system performance, including persons – both individuals and teams (e.g., teacher, students, classroom unit), task(s) (e.g., in-class work, recess play), tools and technologies (i.e., objects that people use to do work or that assist people in doing work), organizational conditions (e.g., schedules, policies, resources), the internal (physical) environment (e.g., physical layout and available space) and external environment. It uses a human factors approach to identify safety threats among the interactions of the system components. The deliberate inclusion of persons emphasises that systems should work to support individuals.

Coding will be both deductive, based on themes from the SEIPS analytic framework, and inductive, looking purposefully for potential safety threats that fall outside of the prescribed framework. If a priori subthemes are not sufficient, the framework may need to be refined or expanded to include new subcategories that lie under the six main SEIPS framework categories. If new sub-themes are identified, further rater training and calibration will be undertaken to ensure acceptable inter-rater reliability around those subthemes. All the codes identified can be identified more than once. Severity of each safety threat will be described using the quality improvement harm scoring system (potential to cause harm; likely to cause minimal harm, likely to cause significant temporary harm, likely to cause permanent harm and death). Additionally, each event will be categorized using the Human Factors Analysis and Classification System as an unsafe act (related to human behaviour), unsafe condition (related to the environment or organisation) or near miss (a situation that involved workers without physical consequence for them).

3.5.5 Study Outcomes: The **primary outcome** will be the number of hand-to-face contacts per hour per student on day 2. This was chosen as the primary outcome for several reasons: 1) measuring actual

infection transmission is not possible in this simulation setting and 2) of the outcomes possible to obtain, it was felt to be most strongly associated with the risk of infection transmission (the most clinically meaningful endpoint).

Secondary outcomes will include:

1. The number of hand-to-mucus membrane contact with eyes, nose or mouth per hour per student
2. The number of hand-to-face contacts with skin or membrane contact per hour per student (excluding isolated mask contact)
3. Proximity contact (being within 1 metre and 2 meters of another person)
4. The proportion of the class (including students and teacher) who were not tagged with the bio-tracer but have dye on their hands and/or face by the end of the day. It is acknowledged that this measure focuses on contact and fomite transmission and will not assess the protective impact of masks for droplet source control or protection. However, it will assess whether mask wearing leads to increased risk of fomite transmission, thereby quantifying potential "harms" from masks.
5. The presence of fluorescein dye on the teacher
6. The number of high touch surfaces in the classroom contaminated with bio-tracer at the end of the day. High touch surfaces in the classroom will include desks, chairs, light switches, classroom door and door handles, cell phones (as appropriate) and any common objects. Surfaces will be identified *a priori* in the classroom and marked as contaminated (dye in any location) or not contaminated (no dye) at the end of the day.
7. The number of interpersonal hand holding per hour per student
8. The number of interpersonal physical contact per hour per student
9. The number of hand hygiene episodes per hour per student
10. The number of mask removals per student per hour (in masking arm only). Masks for source control would only be effective if worn properly. As such, we will quantify the duration of appropriate mask wearing to help balance the risks / benefits.
11. Self-reported experiences of students and teachers in a qualitative substudy (e.g., anxiety, mental health)
12. Human Factors influencing behaviours

3.6 Material Transfer / data linkage and management:

3.6.1 Computer Systems: Personal health information (PHI) required for testing will be collected using REDCap. REDCap is the Hospital for Sick Children's research-focused electronic data capture system REDCap, under an agreement with the software's development consortium, led by Vanderbilt University. REDCap supports two secure, web-based applications designed exclusively to support data capture for research studies. REDCap is a PHP web application served by Apache Tomcat over a 128-bit SSL connection using a signed certificate. The application relies on a study-specific data dictionary defined *a priori* by the investigation team. The data dictionary is the foundation for custom extract from the electronic health record and validation of coding of variables. Authentication of research staff will be performed via LDAP using HSC's enterprise Active Directory service. The application generates a complete audit trail of user activity, provides reporting, and has an automated export mechanism to common statistical packages (SAS, SPSS, Stata, R/S-Plus).

The REDCap MySQL database is replicated in real time to a completely redundant instance of MySQL. The redundant instance is available for restoration of the primary database or for manual failover in the case of primary database failure. Time-stamped backup files are made from the replicated database daily by HSC Research Information Systems using automated backup routines. Backup files are encrypted and transferred to a secure file server accessible only to designated personnel. A rolling seven-day window of

backup files is maintained in an immediately available online state, with a larger window maintained in a compressed file archive

3.6.2 Confidentiality of Subjects: To protect the privacy and confidentiality of the participants, the video data will be deleted after six months. We estimate it will take one-two weeks to de-code the video data and the extra time provides a small safe guard should the video need to be revisited during analysis or manuscript preparation. Audio recordings from focus groups will be retained for six months and then deleted.

3.6.3 Physical Security: All server hardware is securely located in a dedicated secure area. Physical access to the data center is monitored, logged, controlled, and limited to approved personnel. System administration and support is provided by HSC Research Information Systems. Servers and applications are actively monitored 24x7x365 and replicated between two geographically separate, fully redundant data centers. Server backup at the file system level is performed multiple times per day, and where appropriate for volatile data, a multi-day real-time accessible rolling archive can be made available. System and data recovery are accomplished through documented standard operating procedures. Virtual machines will be configured with storage provided through HSC Research Information Systems' enterprise storage solution.

3.6.4 Virtual Environment Security: HSC has made a significant investment in an enterprise-wide VMWare virtualized environment. This technology allows multiple "virtual" machines with potentially different operating systems to be hosted on the same physical server hardware. This infrastructure greatly simplifies monitoring, reduces server administration overhead, promotes cost-effective and efficient use of computing resources, and allows for future flexibility and enhanced capacity with minimal disruption.

The virtualization technology used by VMWare completely separates virtual machines running on the same hardware. Each virtual machine ("guest") instance is completely partitioned into its own sandbox and is unable to interact with or see other guests running on the same physical machine. Access to administer the VMWare environment is limited to authorized HSC Research Information Systems personnel.

3.6.5 Operating System, Database, and Storage Security: All application software is deployed to virtual machines running CentOS (a variant of Red Hat Linux), or if software requires it, Microsoft Windows Server. HSC Research Information Systems personnel manage the base operating system installation and also run regular scans for vulnerabilities, deploying patches as appropriate if software components are out of date.

HSC implements enterprise authentication via the LDAP protocol using Microsoft's Active Directory service. All operating system level authentication will use the HSC Active Directory service. Passwords must meet or exceed HSC Information Security requirements for length and complexity, and they are required to be rotated on a regular basis. Database account information will be stored in a password vault. Operating system level access would be granted only to study personnel and Research Information Systems administrators. All other individuals would be denied access.

In cases where the operating system hosts an application that must communicate over the open Internet (a website for example), the virtual machine will be deployed to the HSC DMo (a location behind HSC's enterprise firewall which allows limited access via the Internet). HSC Information Security regularly scans virtual machines deployed in the DMo for vulnerabilities and currency of patches, providing reports and follow-up scans to both HSC Research Information Systems system administrators and to application owners. All applications and servers will be subjected to these scans, with appropriate remediation of any vulnerability. The HSC enterprise firewall will be configured to only allow connections on ports required for communication with non-HSC personnel (for example, those required for web access). Any other access (for example, to perform operating system maintenance or modify system configuration files) must occur behind the HSC firewall.

Any system which does not need to be directly accessed via the public Internet (a relational database server for example), will be deployed behind HSC's enterprise firewall that provides security for HSC's clinical and research systems. In addition, each individual virtual machine will have its own firewall enabled by the operating system, and only those ports required for administration or communication with public-facing systems will be open. These steps will ensure that no access is possible for non-STUDY individuals.

Data at rest will be secured using operating system and/or database-level security. Database-level authentication will be implemented using strong passwords, meeting or exceeding the HSC requirements for password strength. All data will be stored using the HSC enterprise storage solution managed by Research Information Systems.

3.6.6 Data Collection and Management*:* Data will be uploaded to the SDB through a secure web portal. Data will be stored on a password encrypted HSC computer and access will be limited to HSC investigators and HSC database managing staff. Participating institutions may enter data into the registry using a web interface following approval by the HSC IT team. The Redcap Software will be programmed to remove institution identifiers when aggregate data is released.

Self-reported experiences of students and teachers will be collected using both qualitative and quantitative research methods. Students (> grade 4) and teachers will be asked several open-ended questions post-simulation to gather feedback on the learning environment and their experience with the mitigation strategies. For students, these questions will be posed in-person to each class as a group at the end of the second day of school. For teachers, these questions will be posed during a virtual focus group conducted via ZOOM Healthcare or Microsoft Teams, at the end of the second day of school. These interviews / focus group will be conducted by research staff with training in qualitative methods and will follow an interview guide (see attached). The interviews / focus group will be audio-recorded. Students and teachers will also complete a post-simulation REDCap form.

3.6.7 Future Use of Data: Data will be not be released to third parties. Only the final results of the study (i.e. aggregate data / results) will be provided to the school and non-SickKids study team members.

3.6.8 Risk Assessment: The primary risk of this registry is breach of confidentiality. The study investigators are taking several steps to minimize this risk

- Data will be transmitted and stored securely and in an encrypted fashion online
- Hard data will be stored in locked cabinets in the co-PIs, SickKids offices in rooms 7253 Black Wing and 2406 Black Wing
- Only a small group of investigators will have access to the data set

3.7 Statistical Analysis: We will use a mixed linear regression model adjusted for age and sex with a random intercept for class to analyse the primary outcome. 95% confidence intervals will be bootstrapped. Subgroup analyses planned *a priori* included sex, ethnicity, school type (private vs. public), no mask recommendation (JK – Gr 4 vs. Gr 5 – 12).

Secondary outcomes: We will use appropriate mixed regression models adjusted for age and sex with a random intercept for class to analyse different secondary outcomes. No adjustments for multiplicity will be made for the analysis of secondary outcomes.

Analysis of video: All identified codes (See "video codes") will be charted onto a framework matrix, mapped and open for review by the study team. This matrix is a table that organizes codes by the group (i.e., mask versus non-masked classrooms) in which they occur and by safety threat theme to enable comparison within and across groups. The nature, severity and number of safety threat detected using the SEIPS framework will then be compared across study groups (i.e., mask versus non-masked classrooms)

both descriptively and quantitatively. Summary descriptive statistics will be generated for the number of total safety threats identified per group and time observed. The number of safety threat detected across detection modalities will also be compared using bivariate and regression analyses to identify influencing factors and will be included as co-factors in primary outcome

Proximity data: Proximity data will be summarized in cumulative number and duration per participant and group and included as a co-factor in the primary outcome.

Qualitative data: The audio-recorded interviews / focus group will be transcribed verbatim for qualitative thematic analysis using a qualitative description approach. All data will be read and analyzed by multiple members of the research team. Deductive thematic analysis will be utilized and codes will be developed through line-by-line review of the interviews / focus group discussion using NVivo. Through this review process, codes emerging repeatedly in various parts of the transcript will be noted and comparatively analyzed by multiple research team members for purposes of consistency.

3.8 Sample size: Assuming an intra-cluster correlation coefficient of 0.01, a standard deviation of the number of hand-to-face contacts per hour per student of 6, and a minimal class size of 15 students, we estimate that 12 classes randomized in a 1:1 ratio to either the experimental or to the control arm, will result in more than 80% power to detect an increase in the number of hand-to-face contacts per hour per student from an average of 30 in the control arm to an average of 33 in the experimental arm at a two-sided alpha of 0.05.

The design will be adaptive using an appropriate sequential design. The information size (corresponding to the sample size required to achieve 80% power to detect a difference of 3 hand-to-face contacts per hour per student between experimental and control arm) will be estimated after the first round of simulations based on the actually observed standard deviations, intra-cluster correlation coefficient and the number of hand-to-face contacts per hour per student in the control arm. If the first round of simulations reached the information size, or if pre-specified monitoring boundaries for either a difference or for equivalence are crossed after the first round of simulations, the trial will be stopped. If neither the information size was reached nor a boundary crossed, we will perform a second round of simulations increasing the sample size as appropriate to reach conditional power of 80% under the alternate hypothesis given the accumulated data.

3.9 Timelines: See attached Flow diagram

3.10: Ethical Considerations:

1) Teacher and student consent and assent (where appropriate) will be obtained by the Hospital for Sick Children.
2) While consent will be clear that the purpose is to monitor personal behaviours, parents and students will be kept unaware to specific primary and secondary hypotheses to minimize the Hawthorne effect.
3) Risk of infection: As we will be bringing a group of students and staff together, there is a risk that it could lead to transmission of COVID-19.
    a. Mitigation plan: all children and teachers will be tested for COVID-19 the day prior to the simulation to reduce the risk of infection. Common play spaces will be cleaned throughout the day as per planned implementation in September.
    b. Daily screening for symptoms will occur as per the current Ministry of Education return to school recommendation.
4) Emotional risk for children: it is important to acknowledge that school was stopped abruptly and children were told that it wasn't safe. Preliminary data to date suggests, however, that children's

worsened mental health in Ontario is as a result of public health emergency measures including school closures, rather than as a result of COVID-related anxiety (COVID-19 and Kids Mental Health in Ontario preliminary data, REB#1000070222). Nonetheless, some children may have anxiety upon return to school and/or in participating in the simulations.

   a. Mitigation plan: mindfulness will be included as part of the classroom teaching curriculum on the day of the simulations.
   b. Mask-wearing for a prolonged period of time may also be anxiety provoking or frustrating. A student in the masking arm can refuse to wear the mask at any point and this would be included as a study outcome and they would remain in the study.
   c. Parents will be provided with the mental health resources should they feel that additional mental health support for their child is required. Information will be contained in the "back to school package".

5) Data Handling: All data and records generated during this study will be kept confidential in accordance with Institutional policies and HIPAA on subject privacy. Safeguards will be described outlined in the Data Collection and Management procedures. All video data will be permanently deleted once data coding has been performed. This will be done within 72 hours and will be verified as per the Data Collection and Management procedures
6) Oversight team: A study steering committee will oversee and ensure smooth operation of the study

3.11 Knowledge Translation: We have drawn on our team's clinical, epidemiological, public health and policy-relevant research experience to shape the research question and study design, ensuring that our findings will be of maximal values to the individual, schools and public health. The goal of our knowledge translation activities is to inform policies and practices related to return to school.

REFERENCES

1. American Academy of Pediatrics. COVID-19 Planning Considerations: Guidance for School Re-entry. 2020. https://services.aap.org/en/pages/2019-novel-coronavirus-covid-19-infections/clinical-guidance/covid-19-planning-considerations-return-to-in-person-education-in-schools/ - .XvZQ5fK-xNs.email (accessed July 4, 2020.

2. Canadian Pediatric Society. Supporting a Safe Return to School for Canada's Children and Youth. 2020. https://www.cps.ca/uploads/advocacy/Supporting_a_Safe_Return_to_School.pdf (accessed July 4, 2020.

3. Ministry of Education. Approach to Reopening Schools for the 2020-2021 School Year. Available at http://www.ontario.ca/page/approach-reopening-schools-2020-2021-school-year. (Access Date July 2, 2020).

4. Public Health Ontario. COVID19 - What We Know So Far About…. Infection in Children. Updated May 15, 2020. Available at: https://www.publichealthontario.ca/-/media/documents/ncov/what-we-know-children-feb-21-2020.pdf?la=en.

5. CDC COVID-19 Response Team. Coronavirus Disease 2019 in Children - United States, February 12-April 2, 2020. *MMWR Morb Mortal Wkly Rep* 2020; **69**(14): 422-6.

6. Stokes EK, Zambrano LD, Anderson KN, et al. Coronavirus Disease 2019 Case Survelilance - United States, January 22 - May 30, 2020. MMWR. Available at: https://www.cdc.gov/mmwr/volumes/69/wr/mm6924e2.htm?s_cid=mm6924e2_w (Accessed June 15). 2020.

7. Public Health Agency of Canada. Epidemiological summary of COVID-19 cases in Canada. Last update June 15, 2020. Available at: https://health-infobase.canada.ca/covid-19/epidemiological-summary-covid-19-cases.html - a3.

8. Ludvigsson JF. Children are unlikely to be the main drivers of the COVID-19 pandemic - a systematic review. *Acta Paediatr* 2020.

9. Davies NG, Klepac P, Liu Y, et al. Age-dependent effects in the transmission and control of COVID-19 epidemics. Nat Med (2020). https://doi.org/10.1038/s41591-020-0962-9.

10. Lavezzo E, Franchin E, Ciavarella C, et al. Suppression of a SARS-CoV-2 outbreak in the Italian municipality of Vo'. *Nature* 2020.

11. Ludvigsson JF. Systematic review of COVID-19 in children shows milder cases and a better prognosis than adults. *Acta Paediatr* 2020; **109**(6): 1088-95.

12. Dong Y, Mo X, Hu Y, et al. Epidemiology of COVID-19 Among Children in China. *Pediatrics* 2020; **145**(6).

13. Zimmermann P, Curtis N. COVID-19 in Children, Pregnancy and Neonates: A Review of Epidemiologic and Clinical Features. *Pediatr Infect Dis J* 2020; **39**(6): 469-77.

14. Whittaker E, Bamford A, Kenny J, et al. Clinical Characteristics of 58 Children With a Pediatric Inflammatory Multisystem Syndrome Temporally Associated With SARS-CoV-2. *JAMA* 2020.

15. Feldstein LR, Rose EB, Horwitz SM, et al. Multisystem Inflammatory Syndrome in U.S. Children and Adolescents. *N Engl J Med* 2020.

16. Dufort EM, Koumans EH, Chow EJ, et al. Multisystem Inflammatory Syndrome in Children in New York State. *N Engl J Med* 2020.

17. Shekerdemian LS, Mahmood NR, Wolfe KK, et al. Characteristics and Outcomes of Children With Coronavirus Disease 2019 (COVID-19) Infection Admitted to US and Canadian Pediatric Intensive Care Units. *JAMA Pediatr* 2020.
18. Gotzinger F, Santiago-Garcia B, Noguera-Julian A, et al. COVID-19 in children and adolescents in Europe: a multinational, multicentre cohort study. *Lancet Child Adolesc Health* 2020.
19. Posfay-Barbe KM, Wagner N, Gauthey M, et al. COVID-19 in Children and the Dynamics of Infection in Families. *Pediatrics* 2020.
20. Munro APS, Faust SN. Children are not COVID-19 super spreaders: time to go back to school. *Arch Dis Child* 2020; **105**(7): 618-9.
21. National Institute for Public Health and the Environment. Children and COVID-19. Available at https://www.rivm.nl/en/novel-coronavirus-covid-19/children-and-covid-19. (Accessed July 5, 2020). 2020. https://www.rivm.nl/en/novel-coronavirus-covid-19/children-and-covid-19.
22. Yung CF, Kam KQ, Nadua KD, et al. Novel coronavirus 2019 transmission risk in educational settings. *Clin Infect Dis* 2020.
23. Heavey L, Casey G, Kelly C, Kelly D, McDarby G. No evidence of secondary transmission of COVID-19 from children attending school in Ireland, 2020. *Euro Surveill* 2020; **25**(21).
24. National Centre for Immunisation Research and Surveillance (NCIRS) NSW Government. COVID-19 in schools – the experience in NSW. Prepared by the National Centre for Immunisation Research and Surveillance (NCIRS). April 2020 Report. Available at: http://ncirs.org.au/covid-19-in-schools.
25. Fontanet A, Grant R, Tondeur L, et al. SARS-CoV-2 infection in primary schools in northern France: A retrospective cohort study in an area of high transmission. *Preprint Article: Available at* https://wwwmedrxivorg/content/101101/2020062520140178v2 2020.
26. National Institute for Public Health and the Environment (Netherlands). Children and COVID-19. Available at: https://www.rivm.nl/en/novel-coronavirus-covid-19/children-and-covid-19 (Accessed June 11, 2020).
27. Center for Global Development. Back to School? Tracking COVID Cases as Schools Reopen. 2020. https://www.cgdev.org/blog/back-school-tracking-covid-cases-schools-reopen (accessed July 4 2020).
28. Financial Times. Isreal battles surge in coronavirus infections in schools. https://www.ft.com/content/224fa625-657c-4ffb-a6a0-a40e04d685b9 (accessed July 4 2020).
29. Centers for Disease Control and Prevention. Public Health Considerations for Reopening Schools During the COVID-19 Pandemic. 2020 Available at https://www.cdc.gov/coronavirus/2019-ncov/community/schools-childcare/schools-decision-tool.html
30. City of Toronto. City of Toronto makes masks or face coverings mandatory in enclosed public spaces. Available at https://www.toronto.ca/news/city-of-toronto-makes-masks-or-face-coverings-mandatory-in-enclosed-public-spaces/ (Access Date: July 2, 2020).
31. Wong CY, Tang CS. Practice of habitual and volitional health behaviors to prevent severe acute respiratory syndrome among Chinese adolescents in Hong Kong. *J Adolesc Health* 2005; **36**(3): 193-200.

32. Uchida M, Kaneko M, Hidaka Y, et al. Effectiveness of vaccination and wearing masks on seasonal influenza in Matsumoto City, Japan, in the 2014/2015 season: An observational study among all elementary schoolchildren. *Prev Med Rep* 2017; **5**: 86-91.
33. Suess T, Remschmidt C, Schink SB, et al. The role of facemasks and hand hygiene in the prevention of influenza transmission in households: results from a cluster randomised trial; Berlin, Germany, 2009-2011. *BMC Infect Dis* 2012; **12**: 26.
