# Supplementary material for: Effect of Wearing a Face Mask on Hand-to-Face Contact by Children in a Simulated School Environment: The Back-to-School COVID-19 Simulation Randomized Clinical Trial
Source: JAMA Pediatr. 2022 Oct 24;176(12):1169–75. doi: 10.1001/jamapediatrics.2022.3833 (PMC9593317; doi:10.1001/jamapediatrics.2022.3833)
Supplement: Supplement 5. — Nonauthor Collaborator [file jamapediatr-e223833-s005.pdf]

Supplemental Online Content: Nonauthor Collaborators

*Indicates required information. Only first name, last name, and suffix will appear in PubMed.

*Group Name(s): Back to School COVID-19 School Study Group							
***First Name and Middle Initial(s)**	***Last Name**	***Suffix (eg, Jr, III)**	**Academic Degrees**	**Institution**	**Location (city, state/province, country)**	**Role or Contribution, eg, chair, principal investigator**	**Group (if more than 1 Group listed in the byline) and/or Subgroup (eg, Steering Committee)**
Upton	Allen		MBBS, MSc, FRCPC	The Hospital for Sick Children	Toronto, Ontario, Canada	Advisor	
Allison D.	Alvares		BSc	The Hospital for Sick Children	Toronto, Ontario, Canada	Research Assistant	
Catherine S.	Birken		MD MSc	The Hospital for Sick Children	Toronto, Ontario, Canada	Advisor	
Ahuva	Brown		MD	The Hospital for Sick Children	Toronto, Ontario, Canada	Research Assistant	
Vanessa L.	Carbone		BSc	The Hospital for Sick Children	Toronto, Ontario, Canada	Research Assistant	
Anne	Christie		MSc	The Hospital for Sick Children	Toronto, Ontario, Canada	Research Assistant	
Maureen E.	Cividino		MD CCFP FCFP DOHS CCBOM CIC	Public Health Ontario	Toronto, Ontario, Canada	Advisor	
Justine H.	Cohen-Silver		MSc MD FRCPC MPH	St. Joseph's Health Centre	Toronto, Ontario, Canada	Advisor	
Ronald D.	Cohn		MD	The Hospital for Sick Children	Toronto, Ontario, Canada	Advisor	
Jennifer	Crosbie		PhD	The Hospital for Sick Children	Toronto, Ontario, Canada	Advisor	
Bruno R.	da Costa		PhD	University of Toronto	Toronto, Ontario, Canada	Statistical Support	
Blossom	Dharmaraj		HBSc,MEd	The Hospital for Sick Children	Toronto, Ontario, Canada	Research Assistant	
Sloane J.	Freeman		MD MSc FRCPC	St. Michael's Hospital	Toronto, Ontario, Canada	Advisor	
Karolina	Gaebe		BMath	The Hospital for Sick Children	Toronto, Ontario, Canada	Research Assistant	
Omar	Hajjaj		N/A	University of Toronto	Toronto, Ontario, Canada	Research Assistant	
Lennox	Huang		MD MBA	The Hospital for Sick Children	Toronto, Ontario, Canada	Advisor	
Sarah	Khan		MD MSc	McMaster Univeristy	Hamilton, Ontario, Canada	Advisor	
Eon	Lee		BSc	The Hospital for Sick Children	Toronto, Ontario, Canada	Research Assistant	
Charlotte	Logeman		MPH	The Hospital for Sick Children	Toronto, Ontario, Canada	Research Assistant	
Sanaz	Manteghi		PhD	The Hospital for Sick Children	Toronto, Ontario, Canada	Research Assistant	
Clara	Moore		MSc	The Hospital for Sick Children	Toronto, Ontario, Canada	Research Assistant	
Shaun K.	Morris		MD MPH	The Hospital for Sick Children	Toronto, Ontario, Canada	Advisor	
Julia	Orkin		MD MS FRCPC	The Hospital for Sick Children	Toronto, Ontario, Canada	Advisor	
Sydney D.	Pelger		BSc	The Hospital for Sick Children	Toronto, Ontario, Canada	Research Assistant	
Lauren	Pickel		BSc	The Hospital for Sick Children	Toronto, Ontario, Canada	Research Assistant	
Soha	Salman		BSc	The Hospital for Sick Children	Toronto, Ontario, Canada	Research Assistant	
Ainslie	Shouldice		N/A	The Hospital for Sick Children	Toronto, Ontario, Canada	Research Assistant	
Rachel	Solomon		MPH	The Hospital for Sick Children	Toronto, Ontario, Canada	Advisor	
Nisha	Thampi		MD MSc	Children's Hospital of Eastern Ontario	Ottawa, Ontario, Canada	Advisor	
Kevin	Thorpe		MMath	University of Toronto	Toronto, Ontario, Canada	Statistical Support	
Anna	Wasiak		BSc	The Hospital for Sick Children	Toronto, Ontario, Canada	Research Assistant	
Jiayin	Xie		N/A	The Hospital for Sick Children	Toronto, Ontario, Canada	Research Assistant	
