# Supplementary material for: Effect of Wearing a Face Mask on Hand-to-Face Contact by Children in a Simulated School Environment: The Back-to-School COVID-19 Simulation Randomized Clinical Trial
Source: JAMA Pediatr. 2022 Oct 24;176(12):1169–75. doi: 10.1001/jamapediatrics.2022.3833 (PMC9593317; doi:10.1001/jamapediatrics.2022.3833)
Supplement: Supplement 6. — Data Sharing Statement [file jamapediatr-e223833-s006.pdf]

Data Sharing Statement

Science. Effect of Wearing a Face Mask on Hand-to-Face Contact by Children in a Simulated School Environment. *JAMA Pediatr*. Published October 24, 2022.
doi:10.1001/jamapediatrics.2022.3833

Data
Data available: Yes
Data types: Deidentified participant data
How to access data: Deidentified participant data is available upon request after publication with a signed data access agreement.
When available: beginning date: 01-01-2023

Supporting Documents
Document types: None

Additional Information
Who can access the data: Researchers whose proposed use of the data has been approved and a data access agreement has been signed.
Types of analyses: For a specified purpose approved by study authors.
Mechanisms of data availability: After approval of proposal and with a signed data access agreement.
